# Patterns of kinesin evolution reveal a complex ancestral eukaryote with a multifunctional cytoskeleton

**DOI:** 10.1186/1471-2148-10-110

**Published:** 2010-04-27

**Authors:** Bill Wickstead, Keith Gull, Thomas A Richards

**Affiliations:** 1 Sir William Dunn School of Pathology, University of Oxford, South Parks Road, Oxford, OX1 3RE, UK; 2 Centre for Eukaryotic Evolutionary Microbiology, School of Biosciences, University of Exeter, Devon EX4 4QD, UK

## Abstract

**Background:**

The genesis of the eukaryotes was a pivotal event in evolution and was accompanied by the acquisition of numerous new cellular features including compartmentalization by cytoplasmic organelles, mitosis and meiosis, and ciliary motility. Essential for the development of these features was the tubulin cytoskeleton and associated motors. It is therefore possible to map ancient cell evolution by reconstructing the evolutionary history of motor proteins. Here, we have used the kinesin motor repertoire of 45 extant eukaryotes to infer the ancestral state of this superfamily in the last common eukaryotic ancestor (LCEA).

**Results:**

We bioinformatically identified 1624 putative kinesin proteins, determined their protein domain architectures and calculated a comprehensive Bayesian phylogeny for the kinesin superfamily with statistical support. These data enabled us to define 51 anciently-derived kinesin paralogs (including three new kinesin families) and 105 domain architectures. We then mapped these characters across eukaryotes, accounting for secondary loss within established eukaryotic groupings, and alternative tree topologies.

**Conclusions:**

We show that a minimum of 11 kinesin families and 3 protein domain architectures were present in the LCEA. This demonstrates that the microtubule-based cytoskeleton of the LCEA was surprisingly highly developed in terms of kinesin motor types, but that domain architectures have been extensively modified during the diversification of the eukaryotes. Our analysis provides molecular evidence for the existence of several key cellular functions in the LCEA, and shows that a large proportion of motor family diversity and cellular complexity had already arisen in this ancient cell.

## Background

The transition from prokaryote to eukaryote was a hugely important event in the evolutionary history of life and provided the foundations for the evolution of numerous complex organismal forms. Present day eukaryotes differ fundamentally from prokaryotes in having much higher complexity of cell organization. This complexity cannot have appeared fully-formed, but arose by stepwise elaborations of cell structure - implying that certain lineages of extant eukaryotes might have retained "simpler" ancestral features (see [1,2]). However, the order and relative importance of many of the acquisitions that must have occurred to allow the cellular features now seen in extant eukaryotes remain controversial. By comparing the genomes of a wide taxonomic range of eukaryotes, and including sufficient taxon sampling to account for secondary loss, we can reconstruct the likely genomic composition of the last common eukaryotic ancestor. In this way, it is possible to reconstruct the ancestral repertoire for some of the molecular components of key eukaryotic features and identify evidence for intermediate states, if they exist. This in turn helps us to understand the biology of the ancestral eukaryote and how the prokaryote-eukaryote transition proceeded.

One of the key changes that enabled increased cellular complexity in eukaryotes was the evolution of the cytoskeleton - based ancestrally on actin filaments and tubulin-based microtubules (intermediate filaments most probably only appearing later in a specific lineage). This network and its associated motors, plays an essential role in several eukaryote-defining cellular processes, including division of genetic material at mitosis and meiosis, inheritance of cytoplasmic organelles, intracellular transport of vesicles, and cellular motility based on either crawling or beating of cilia/flagella. In keeping with this central role, cytoskeletal motor proteins arose early in the eukaryotic lineage [3-5]. Of the three superfamilies of motors - kinesins, dyneins, and myosins - only the kinesins are ubiquitous to all eukaryotes thus far analyzed [6-9]. To shed light on the cellular complexity of the last common eukaryotic ancestor, we analyzed the kinesin motor protein superfamily using comparative genomics, protein domain architecture analysis and the most comprehensive supported kinesin motor domain phylogeny to date. From these data, we look at the evolution of the kinesin superfamily across eukaryotes. We also reconstruct the kinesin repertoire of the LCEA and infer some of the biological features of this ancestral cell.

## Results and Discussion

### Diversification of kinesin paralog families

To map the ancient evolutionary history of the kinesin gene family we surveyed 45 eukaryotic organisms for which complete or near-complete genome was publicly available. These organisms represent a wide taxonomic diversity of eukaryotes and encompass five of the six proposed eukaryotic 'supergroups' [10,11]. To survey for kinesins, we used a hidden Markov model-based strategy [12] using the Pfam kinesin motor domain model (PF00225; see Material and Methods for details). This approach identified 1624 encoded kinesin-like protein sequences (Additional file 1). To improve phylogenetic resolution and analysis speed we removed 166 sequences with scores <100 (expectation value > 10^-25^), representing the most divergent kinesin-like sequences. This threshold is lower than used in previous work [8] and sufficiently liberal to include all the previously identified kinesins from *Schizosaccharomyces pombe *and *Saccharomyces cerevisiae *(including the divergent kinesin Smy1) - and also include all kinesins from *Drosophila melanogaster *except the atypical Cos2 (which may have no motor activity, binding to microtubules in an apparently ATP-independent manner [13]) and all but the very-highly divergent VAB8 (klp5) from *Caenorhabditis elegans*. We aligned the motor domains from these 1458 protein sequences, trimmed the alignment to 330 well-conserved characters and removed 195 near-identical sequences (>95% identity). From this alignment we calculated a Bayesian phylogeny by combining 8 independent runs of MrBayes3.1.2 [14]. To evaluate support for the inferred tree, we used two approximate Likelihood Ratio Test (aLRT) methods [15,16]. These methods estimate support for each node by systematically measuring the ratio of the likelihood of the given tree to an alternative topology in which that node has been collapsed (see Materials and Methods). We considered as well-supported only those tree topology nodes with p > 0.95 by both aLRT methods. The identities of these well-supported nodes are largely independent of the amino-acid substitution matrix used in the test (see Material and Methods).

**Figure 1:**
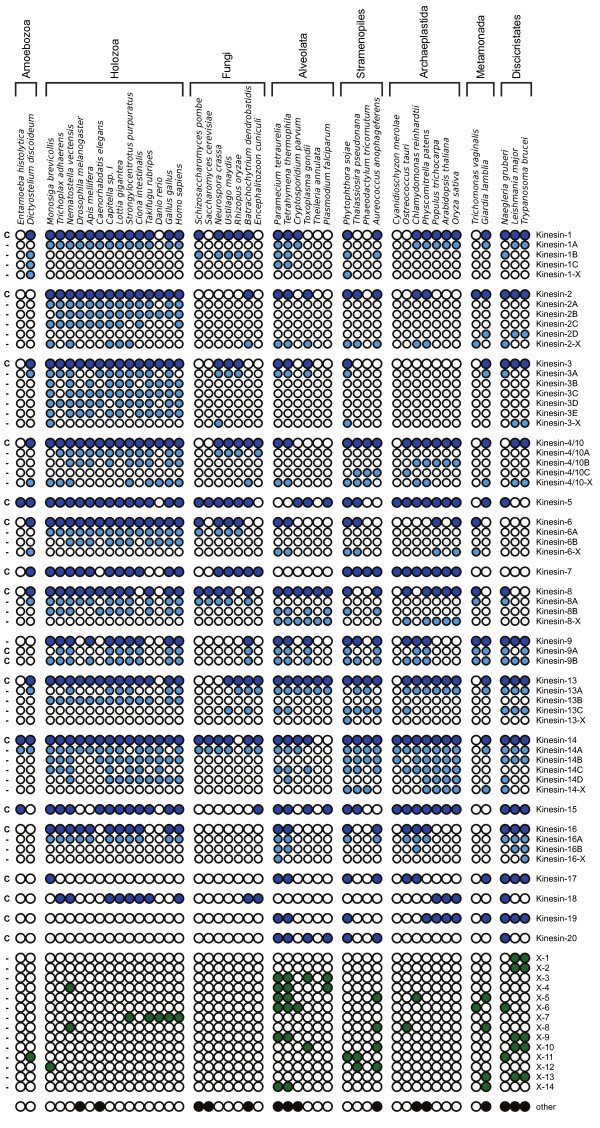
**Distribution of ancient kinesin paralogs in 45 diverse eukaryotes**. Using the results of our comprehensive kinesin motor domain phylogeny (Additional file 2) we identified 51 kinesin paralogs, encompassing 17 kinesin families and 34 subfamilies. Presence of paralog(s) in a genome is indicated by a filled circle, absence/not-found is indicated by an open circle. Only paralogs from well-supported nodes were considered (p > 0.95 by both aLRT methods; see Additional file 2). Dark blue circles indicate presence of members of a full kinesin family (corresponding to the deepest well-supported nodes for kinesin groups containing sequences from eukaryotes belonging to more than one eukaryotic "supergroup"), whilst subfamily paralogs are indicated by light blue circles beneath (suffixed A, B, C etc.). Kinesins falling within a particular kinesin family, but outside of all the contained well-supported subfamilies are suffixed '-X' (e.g. Kinesin-1-X). Groups of kinesins that do not have sufficient membership to be considered full kinesin families (see Results and Discussion) are numbered X1 to X14 (green circles). Species analyzed are grouped into higher taxonomic groups. Paralog families used in Dollo parsimony analyses are marked 'c' (character) adjacent to the first column.

Additional file 2 contains a 1263-sequence Bayesian phylogeny for the kinesin repertoires encoded by the 45 diverse eukaryotes. Each of the 14 kinesin families defined previously by Wickstead and Gull [8] in a smaller analysis of 19 genomes (i.e. Kinesin-1, 2, 3, 4/10, 5, 6, 7, 8, 9, 13, 14, 15, 16, and 17) were also retrieved here with strong topology support (>0.95 by both aLRT methods). In addition, based on the criteria set out by Lawrence et al. [17], our analysis supports the existence of three new kinesin families, which we name Kinesin-18, 19, and 20 - to follow on from previously identified families (Figure 1 and Additional file 2). Each of these new kinesin families has strong support and a wide taxonomic distribution amongst the eukaryotes sampled. As in previous work [8], in this extensive phylogeny - which includes full kinesin repertoires from a broad range of eukaryotes - we find no support for kinesin families -10, 11, or 12 [17].

Our phylogenetic analysis provided evidence for an additional 14 paralog groups, which were not part of kinesin families on our phylogenetic tree. Each of these paralog groups was well supported, but none are considered bona-fide kinesin families at this stage, either because they lacked sufficient membership (<1% of sequences examined) or contained only sequences from one eukaryotic supergroup. We designated these additional tentative paralog families X1-X14 (Figure 1 and Additional file 2). Names, unique identifiers and kinesin family/subfamily for all the 1624 identified kinesins in this study can be found in Additional file 3.

By definition, each kinesin family is shared by at least two eukaryotic supergroups [17] and is therefore most likely anciently derived (although not necessarily ancestral). In addition to these families, our analysis shows that there are multiple paralogs within at least 10 kinesin families (Kinesin-1, 2, 3, 4/10, 6, 8, 9, 13, 14 and 16; Figure 1) that are most likely the products of additional ancient gene duplication events. In keeping with the standardized nomenclature of Lawrence et al. [17] we have identified well-supported subfamilies by appending a letter to the family name (e.g. Kinesin-9A and 9B). In this analysis we have considered two levels of "ancient" paralogy: 1) well-supported kinesin families shared by at least two eukaryotic supergroups, and also 2) subfamilies for which there is evidence at least for the paralog being present at the root of a major taxonomic group (with the exception of Kinesin-2B, for which only the metazoan members form a well-supported clade, but for which there is a probable ortholog in *Monosiga*; see Additional file 2). All subfamilies have good topological support (p > 0.95 using both aLRT methods, as above).

The identification within several kinesin families of paralogs shared by multiple eukaryotic supergroups suggests that the use of family name alone does not accurately reflect the evolutionary (or functional) complexity of the kinesin motor families. Our analysis suggests that the evolutionary diversification of the kinesin gene family has been extremely complicated, encompassing at least 51 ancient paralogs (Figure 1). The majority of these paralog forms arose from gene duplication events that at least predate the major taxonomic units of eukaryotes [10,11] and therefore most likely arose in an early phase of eukaryotic evolution. It is worthy of note that our phylogeny (Additional file 2) also shows evidence of paralogs in closely related organisms that are the result of relatively recent lineage-specific duplication events. These paralogs are not the focus of this work and will not be discussed at length here, but they demonstrate that kinesin diversification is not restricted to events very early in eukaryotic evolution and gene duplication has generated novel kinesin genes throughout the diversification of the eukaryotes.

### Diversification of kinesin protein architectures

Motor proteins are generally composed of a motor head domain that converts chemical energy to force, and a range of additional domains that bind cargo, filaments or accessory proteins (e.g. [18,19]). Since regions outside of the motor head domain direct many interactions, considerable functional diversification might be achieved through the evolution of the protein domain combinations. To further investigate the diversification of the kinesin superfamily, we identified putative domain architectures for all 1624 identified kinesin proteins using Pfam and CDD database searches [20,21]. In total we found 105 different kinesin protein domain architectures (Additional file 4; domain architectures for all 1624 identified kinesins are available in Additional file 3). Surprisingly, most domain architectures were specific to only one organism in our analysis, indicating that these domain combinations were relatively recent acquisitions. It is also noteworthy that most kinesins in our analysis (1300/1624) possess no identifiable protein domains outside of the motor itself. This implies that the great majority of the interactions between these motors and other proteins is controlled either by poorly conserved stretches of peptide or protein domains that are not yet described in protein domain databases.

**Figure 2:**
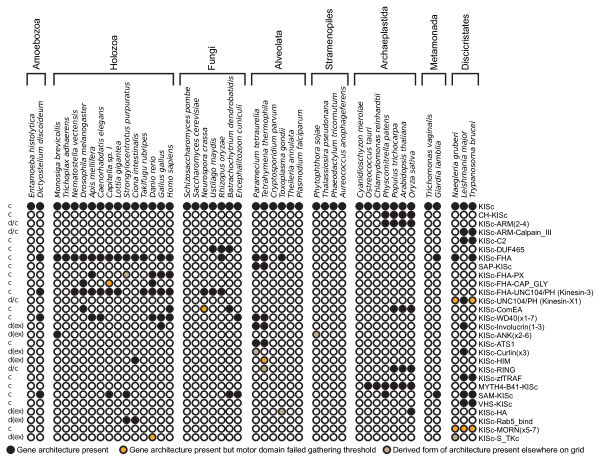
**Distribution of kinesin protein architectures in 45 diverse eukaryotes**. Pfam and CDD searches were used to identify putative gene architectures for the 1624 kinesin proteins identified in the genome datasets. All unique gene architectures identified in two or more genomes are shown here while all 105 different gene architectures identified are shown in Additional file 4. Presence of a gene architecture in a genome is indicated by a filled circle, absence/not-found is indicated by an open circle. Species analyzed are grouped into higher taxonomic units. Architectures used in Dollo parsimony analyses are marked 'c' (character), while architectures, which appeared not to be homologous based on further investigation (see Additional file 6), are marked 'd(ex)' (discounted and excluded), while this analysis adjusted the taxon distribution of some architecture characters marked 'd/c' (discounted and corrected) adjacent to the first column. Domains found more than once are numbered to indicate the multiples in which the domains are found (e.g. x2-7 indicates the protein contained between 2 and 7 copies of the domain).

Of the 105 kinesin domain architectures, 28 are found in two or more genomes suggesting an origin predating the last common ancestor of the species that possess this specific domain architecture (the distribution of these is shown in Figure 2). By annotating the motor domain phylogeny with the protein domain architectures (Additional file 2) it is possible to identify cases where different architectural forms are the result of secondary loss of domains (e.g. Kinesin-3D family KIF13B orthologs from human and chicken lack the CAP_GLY domain). Accounting for these secondary loss events, 21 protein domain architectures that were found in multiple genomes were specific to a paralog or family on the kinesin phylogeny, suggesting that they represent derived character states (Additional file 5). However, in several cases the phylogeny suggested that the similar protein domain architectures occupied very distant branching positions in the kinesin phylogeny, and were absent from all species that occupied intermediate branches. We investigated this further by comparing the results of Pfam and CDD searches and aligning the relevant protein domains. In 7 cases we found no convincing alignment between the domains suggesting that these features are not homologous. These domain architectures were therefore excluded from further analysis (Additional file 6; marked 'd(ex)' on Figure 2). In a further 4 cases, following the same principle, we corrected the taxon distribution of a specific domain architecture because the domain found connected to the kinesin motor did not appear to be homologous to the other protein sequences included in that architecture type (Additional file 6; marked 'd/c' on Figure 2).

After the exclusion of unreliable and convergent kinesin architectures (Additional file 6), a total of 21 architectures were identified that potentially represent shared derived characters. These 21 characters (see Additional file 5; marked 'c' on Figure 2), were included in our analysis of kinesin protein evolution (below). Several of these domain combinations are widely distributed among the species analyzed, suggesting that the protein domain architecture had an ancient ancestry within the eukaryotes and that shuffling of protein domains linked to the kinesin motor has played an important role in the early diversification of many kinesin protein families.

### The kinesin repertoire of the last common eukaryotic ancestor

**Figure 3:**
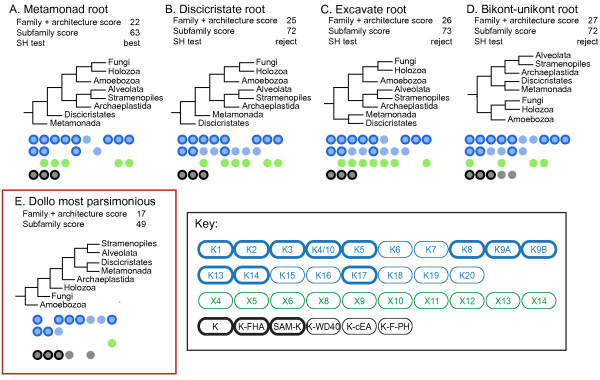
**Defining the kinesin repertoire of the last common eukaryotic ancestor (LCEA)**. We considered 5 rooted eukaryotic trees to infer conservative estimates of the minimal ancestral repertoire of kinesins present in the LCEA using Dollo parsimony: **A**) 'Metamonada-first'; **B**) 'Discicristata-first'; **C**) 'Excavata-first'; **D**) root between unikonts and bikonts; **E**) Dollo most parsimonious tree necessary to explain the extant distribution (boxed in red). The unconstrained most parsimonious tree gives an unrealistic eukaryotic tree topology and therefore is likely to underestimate the LCEA repertoire (see Results and Discussion). The parsimony scores under the two alternative datasets are shown for all 5 topologies. Also shown are the results of SH alternative topology tests for the four alternative models under the polytomy favored by the analysis of Burki et al. [26] (see Results and Discussion). Paralog families and kinesin architectures, which must have been present in the LCEA given the tree topology are shown beneath each tree. Kinesin paralogs are colored blue for families (K1-20) and green for non-families (X1-14; see Figure 1). Kinesin protein domain architectures are shown in black (see Figure 2). These analyses indicate minimally 18 to 29 kinesin characters (paralogs/architectures) in the LCEA. Kinesin characters present in the LCEA under the 4 leading models of the eukaryotic tree topology, A-D (the *minimal ancestral repertoire *- MAR) are marked in bold.

To investigate the minimum complement of kinesin forms present in the LCEA, we mapped the ancestral repertoire of kinesin characters under four alternative eukaryotic evolutionary trees (Figure 3A-D). We coded the presence and absence of kinesin families (marked 'c' Figure 1) and reliable protein architectures (marked 'c' and 'c/d' Figure 2) as binary characters. In both cases, these selections included characters that were strongly suggested to be monophyletic (see discussion above). To further ameliorate patterns of secondary loss we coded the presence and absence of kinesin paralogs and architectures by combining the species data into 8 higher taxonomic groups (as marked on Figures 1 and 2). These taxonomic groups are based upon those recovered in several multi-gene phylogenies [22-26], which have demonstrated a consensus higher level grouping of the eukaryotes. At least 2 of the suggested supergroups within eukaryotes (Excavata and Chromalveolata) remain contentious [22]. To control for this we only used sub-groupings within Excavata and Chromalveolata that are currently strongly supported. The combination of paralogs and architectures produced a binary data matrix of 8 'taxa' and 39 characters. To further investigate the ancestral diversification of kinesin gene families we generated an alternative character matrix based on 51 characters produced from only the kinesin subfamily data. We used a Dollo parsimony analysis method [27,28] to investigate the possible branching order of the 8 higher taxonomic units and the minimal ancestral repertoire of kinesin characters present in the LCEA. Dollo parsimony explains the presence of a state by allowing only one genesis event for a character, and as many losses as are necessary to explain the pattern of characters seen [27]. The method makes the assumption that the ancestral state is character absence and therefore generates a tree topology that provides the minimum complement of kinesin types present in the common ancestor of all 45 genomes sampled.

The phylogenetic branching order and root position of the eukaryotes is a contentious issue. Burki et al. [26] have recently performed large-scale phylogenetic analysis of concatenated sequence and suggest three major eukaryotic branches: excavates (which in their analysis included the discicristate group containing *Trypanosoma, Leishmania*, and *Naegleria*) [29], unikonts (containing Metazoa, Fungi and Amoebozoa) [7,30] and a major clade, which encompasses the majority of phototrophic or ancestrally phototrophic eukaryotes (containing Archaeplastida, stramenopiles and alveolates) [26]. Many aspects of these groupings are also consistent with other concatenated multi-gene phylogenetic analysis [22,23,31,32]. The results of the Burki et al. analysis [26], however, did not sample the metamonad genomes (*Trichomonas *and *Giardia*) [33,34], which have also been tentatively classified as excavates [29] (see [22,32,35] for phylogenetic evidence of monophyly if not holophyly), but were excluded because these taxa often produce long branches within phylogenetic trees and are therefore potentially a source of artifact in tree inference [36].

As it has been suggested that the metamonad branch may represent the first branch in the eukaryotic phylogeny and the excavates may be paraphyletic to the root of the eukaryotes, the consensus view of the eukaryote phylogeny is a polytomy of four major clades: 1) metamonads (e.g. *Trichomonas *and *Giardia*); 2) discicristates, (e.g. *Trypanosoma, Leishmania *and *Naegleria*); 3) unikonts (including Metazoa, Fungi and Amoebozoa); and 4) a large 'ancestrally phototrophic' clade (including Archaeplastida, stramenopiles, and alveolates) [26]. Therefore, a number of primary branch groups are possible. We used a Dollo parsimony approach to compare four topological variations possible within this polytomy (Figure 3A-D) with the results of an unconstrained Dollo parsimony analysis (Figure 3E). These alternative topologies included a tree that placed the metamonads (*Trichomonas *and *Giardia*) as the first branch [37-39] and a tree topology equivalent to the bikont-unikont model [7,30,40,41].

For comparison we have included the most parsimonious tree generated when using the Dollo method without any topological constraint (Figure 3E). This is the simplest possible explanation for the extant distribution of the characters if no assumptions are made with regards to the branching order of that tree. The resultant tree is very unlikely to be a realistic eukaryotic phylogeny. However, even given this topology, the LCEA possessed a complex repertoire of minimally 11 ancestral kinesin families and 5 kinesin architectures (Figure 3E).

Each of the 4 likely alternative topologies for eukaryotic evolution implies a slightly different ancestral kinesin repertoire in the LCEA (Figure 3A-D). However, our analysis identifies a complex core set of ancestral characters that were present in the LCEA under any of these 4 tree topologies. These include 11 kinesin paralogs - namely, Kinesin-1, 2, 3, 4/10, 5, 8, 9A, 9B, 13, 14, 17 - and 3 protein domain architectures - KISc, KISc-FHA, SAM-KISc. This core set will here be referred to as the *minimal ancestral repertoire *(MAR and are marked in bold on Figure 3). These results show that a large proportion of the extant diversity of the kinesin superfamily was already established before the radiation of eukaryotes from the LCEA. They also strongly suggest that the ancestral eukaryotic cell had a complex biology built around a microtubule-based cytoskeleton.

It is clear that several kinesin families are linked to specific cellular functions [17,42]. However, for some families pleiotropy and a lack of knowledge of function across a broad taxonomic base makes it difficult to unambiguously infer ancestral function. Of the 11 paralog families in the MAR, at least three have conserved functions in nuclear division (mitosis and/or meiosis; Kinesin-5, -13 and -14) that are most likely ancestral to the whole family. From this, we can infer that the LCEA built a bidirectional spindle containing both plus-end directed (Kinesin-5) and minus-end directed (Kinesin-14) motors [43-46]. The presence of these antagonistic motors suggests that, even in early eukaryotic cells, spindle construction relied on generation of counteracting pole-to-pole forces (see [47-49]). Alongside these spindle motors, the LCEA encoded a Kinesin-13 microtubule depolymerizing motor [50,51], possibly embedded in the kinetochores, as it is in several extant species examined [52-55]. It is credible to suggest that the Kinesin-8 and Kinesin-4/10 (also part of the MAR) were also part of this ancestral nuclear division mechanism. However, the identification of significant alternative roles for these families outside of nuclear division [56-58], makes the possibility of this being the ancestral function tentative.

The presence of Kinesin-1 and -3 paralogs in the MAR, suggests strongly that the LCEA had the capacity to traffic membrane-bound bodies within the cytoplasm [59-63]. This implies that the ancestral cell built cytoplasmic microtubules and processed vesicular traffic - in agreement with the wide taxonomic distribution of many additional components of the eukaryotic membrane-trafficking system in extant eukaryotes [64].

Notably, none of the four trees representing alternative hypotheses encompassed by the eukaryotic ancestral polytomy model represents the most parsimonious topology under the Dollo approach. The most parsimonious explanation of the observed data (Figure 3E) is clearly inconsistent with any current views of the eukaryotic branching order. The placing of Amoebozoa as the primary eukaryotic branch is almost certainly an artifact caused by the lack of flagella/cilia and the associated loss of kinesins with ciliary function in the two amoebozoa for which complete genome data is publicly available. Such artifact has been described previously [8]. Consistent with the hypothesis that the positioning of the Amoebozoa in unconstrained trees is an artifact of ciliary loss, the ancestral repertoire implied by the most parsimonious unconstrained tree is the MAR set without the families associated with cilia/flagella [8] (Kinesin-2, 9A, 9B and 17; Figure 3E/F). We investigated what evidence for kinesin paralogs might be available from expressed sequence tag sequencing of amoebozoan organisms which build flagella. However, only 2 and 1 kinesin motor fragments are contained in the expressed sequence tag libraries for *Mastigamoeba *and *Hyperamoeba*, respectively from TBestDB [65]. Of these, the *Mastigamoeba *sequences could be placed with reasonable confidence into the Kinesin-14A and Kinesin-13 groups (the fragment of kinesin sequence from the two *Hyperamoeba *datasets could not be grouped; data not shown).

Finally, the MAR, as defined by comparison of 4 alternative eukaryote topologies above, shows that the LCEA had a cilium/flagellum. Kinesin-2 is the anterograde motor of the intraflagellar transport (IFT) machinery - a series of components critical for building and servicing cilia/flagella (see [66,67]). In *Chlamydomonas*, the protein KLP1 (Kinesin-9A) is a part of the central apparatus of the cilia [68], although the level of conservation of this function is yet to be widely assessed. For two of the MAR paralogs - Kinesin-9B and Kinesin-17 - there is currently no published functional data at all. However, the presence of Kinesin-2, 9A, 9B and 17, and also the non-MAR family Kinesin-16, only in organisms which build flagella/cilia at some stage in their lifecycle ([8] and Figure 1) predicts an ancestral role associated with this organelle.

## Conclusions

The microtubule-based cytoskeleton in extant eukaryotes - with its motors and accessory proteins - is vastly more complex than the prokaryotic FtsZ-based system from which it evolved (see [69]). It is used in many of the cellular processes that define eukaryotes. Yet there is little molecular evidence for the timing of the acquisition of several of these key features. Here, we have explored the evolution of the eukaryotic cytoskeleton through the evolution of its kinesin motors. We have used genomic information from 45 diverse eukaryotes to produce the most extensive kinesin phylogeny to date, for which we have derived statistical support. We have used this to define 51 anciently-derived kinesin paralogs, contained within 17 kinesin families and 34 subfamilies. We also defined 105 gene architectures for the 1624 kinesin sequences included in the analysis - of which only 6 architectures are shared between the major taxonomic groups in our analysis.

The branching order of the major lineages of eukaryotes is still a contentious issue. However, by accounting for multiple possible topologies, as well as secondary loss, we have shown that a minimum of 11 kinesin families were present in the last common eukaryotic ancestor. The prevailing trend in current models of early eukaryotic cell evolution is the proposal of stepwise acquisition of cellular complexity with particular extant eukaryotic lineages being identified as derived from intermediary and primitive phases of early eukaryotic evolution (reviewed in [1]). This idea is contradicted by the results presented here, which demonstrate that, at least for the kinesin-driven cytoskeleton, the LCEA already possessed a highly complex cellular form *before *giving rise to any of the sampled extant eukaryotic groups. This proto-eukaryotic cell was surprisingly highly developed in terms of kinesin motor types - containing the majority of families now found in eukaryotes. In contrast, the domain architectures of these motors have been much more extensively modified during diversification of lineages, such that only 3 can be unambiguously traced back to the LCEA. These results are consistent with a growing body of literature which suggests that the LCEA had a highly complex cellular form. Alongside the complex kinesin repertoire shown here, this ancestral cell possessed genes encoding the major cellular components of meiosis [70], a derived and complex DNA replisome [71], and many components required for endocytosis [64,72] and probably phagotrophy [30].

The kinesin types present in the LCEA provide molecular evidence for some of the cellular processes present in the proto-eukaryote. The LCEA had nuclear division machinery that included antagonistic motors to generate tension and kinetochore-associated microtubule depolymerizing agents. It also trafficked vesicles along cytoplasmic microtubules and built an axoneme with a central apparatus (and which, on the basis of dynein distribution, was motile [9]). The data presented here also show that, although there have been significant gene duplication events within the kinesin families (for example deep within the metazoa and also the land plants), the history of kinesins is in many cases a history of paralog loss from an ancestral form which possessed a motor repertoire more complex than many extant organisms.

## Methods

### Kinesin motor domain phylogeny

Predicted protein datasets were obtained for 45 diverse eukaryotes for which complete or near-complete genome sequence data is publicly available. Additional file 7 provides a comprehensive list of sources and versions for these datasets. From these datasets, we extracted complete kinesin repertoires using HMMERv2.3.2 [22] to find all predicted proteins with a match to the Pfam 'kinesin motor domain' profile (PF00225; [69]). In total, 1624 sequences match the kinesin motor model at or above the 'gathering threshold' (score = -135; expectation value < 2 × 10^-4^). However, for phylogenetic reconstructions, highly divergent sequences cause problems with both sequence alignment and tree inference [73] and we found that inclusion of the most divergent kinesin sequences hindered tree reconstruction (data not shown). For this reason, 166 sequences with scores < 100 (expectation value > 10^-25^), representing the most divergent sequences, were excluded from phylogenetic analyses (Additional file 1). The remaining 1458 sequences were trimmed to 80 aa either side of the kinesin motor domain (as defined by the Pfam model) and the motors domains aligned using MAFFT6.24 [74] adopting the E-INS-i strategy [75]. This alignment was then trimmed to well-aligned blocks (330 characters) and we reduced redundancy in the dataset by removing 195 sequences from duplicated genes that encode proteins predicted to be identical or nearly identical (>95% identity at the amino acid level) to other sequences from the same organism. Both untrimmed and trimmed alignments are available in Additional file 8 and 9, respectively.

Bayesian phylogenies were inferred from the protein alignment using metropolis-coupled Markov chain Monte Carlo (MCMCMC) method as implemented in the program MrBayes3.1.2 [14]. The WAG substitution matrix was used [76] with a gamma-distributed variation in substitution rate approximated to 4 discrete categories and shape parameter estimated from the data (mean α = 0.927). Ten runs were preformed each consisting of 4 Markov chains heated to a 'temperature' of 0.2 and run for 12,000,000 generations. All runs were initiated from a starting tree inferred from BLASTp scores as described in [8] - a strategy which gave significantly better stationary phase tree likelihoods than those using starting trees inferred by either maximum parsimony or neighbor-joining (data not shown). Chains were sampled every 8,000 generations. Two runs, which did not reach apparent stationary phase by halfway through the run, were discarded. For the remaining 8 runs, the first 6,400,000 generations of each was discarded as burn-in and the remaining generations were used to construct the majority-rule consensus tree shown in Additional file 2.

### Assessing topological support for the kinesin tree

Since the scale of the phylogenetic analysis (1263 sequences) made bootstrap replication unfeasible, we tested the level of support for the inferred topology using the approximate Likelihood Ratio Test (aLRT) method of Anisimova and Gascuel [15]. Both non-parametric Shimodaira-Hasegawa-like (SH) and parametric χ^2^-based p-values were generated using the aLRT implementation in PhyML 3.0 [16] with the LG substitution matrix [77]. It is likely that both aLRT methods provide a better estimate of branch support than do Bayesian posterior probabilities. aLRT methods directly test the inferred topology by comparing it to an alternative topology where each node has been systematically collapsed. In contrast, Bayesian methods rely on adequate sampling of the posterior distribution of topologies to provide a good estimate of the posterior probabilities. Because our dataset is highly complex and the tree topology was calculated from a very large MCMCMC search, the resulting trees sampled for the consensus tree will include numerous trees with slight variations in topology by virtue of stochastic error within the MCMCMC sampling procedure. This has the effect of increasing the frequency of recovery of low posterior probabilities in large and complex datasets, as is evident when compared to the results of the aLRT topology assessment methods (Additional file 10). Kinesin families (K1-20) were defined as encompassing all sequences within the most basal clans having p > 0.95 support in both aLRT tests. To test the affect of a change in amino acid substitution matrix, we repeated the aLRT test using the WAG [76] and JTT matrices [78]. Of the 485 nodes recovered in the phylogenetic analysis supported with p > 0.95 for both χ^2^- and SH-based approximate likelihood ratio tests using the LG matrix, 461 (94.5%) and 463 (94.9%) were recovered with p > 0.95 for both tests when using the WAG or JTT matrix, respectively - demonstrating that a change in matrix had a relatively minor effect in the clade support values used to classify kinesin paralogues.

Unsurprisingly, the proportion of sequences falling into one of the well-supported kinesin families decreases as the 'quality' (as assessed by Pfam score) of the kinesin motor domain decreases (Additional file 11). This implies that a large proportion of the highly divergent kinesin motors excluded from tree inference do not belong to established kinesin paralog families, and it is unlikely that large numbers of bona fide family members were excluded from our analysis.

### Identifying kinesin protein architectures and ancient patterns of kinesin evolution

We used all 1624 sequences identified from the HMMER search as separate search seeds for PfamA [20] and CDD [21] searches in order to identify the presence and relative order of conserved protein domains. The results of the two protein architecture searches were compared, noting the relative position of the domains within the amino acid sequence. Using these comparisons consensus putative domain architecture were identified for each protein sequence. All architecture types were mapped onto our comprehensive phylogeny in order to identify the phylogenetic distribution of the protein architectures (Additional file 2). Kinesin protein architectures specific to paralog families or specific phylogenetic clusters were judged as the product of a single protein domain rearrangement or domain acquisition event (Additional file 5; see Additional file 6 for exclusions). We identified several kinesin domain architectures, which include domains present in a low number of distantly related genomes or for which the kinesin motor domains belong to distantly related paralog families. In these cases, we conducted further analysis to investigate whether these sequences were composed of domains related by either convergence or vertical inheritance, or if the domain classification was artifactual. For each candidate domain architecture marked 'd' on Figure 2, functional and annotation data was accessed from Pfam and CDD [20,21], domain alignments were made using MUSCLE and manually edited using the SEAVIEW alignment platform [79,80]. 11 cases of domain classification, for which no good evidence of homology could be found, were either excluded as likely artifact or adjusted for taxon distribution as appropriate (Additional file 6). SAM1 and SAM2 domains are homologous and were classified as one domain for the purposes of this study (Additional file 6).

### Evaluating kinesin evolution under alternative eukaryotic tree topologies

To investigate the minimum complement of kinesin forms present in common ancestor of all 45 genomes sampled, we coded the presence and absence of kinesin families (marked 'c' Figure 1) and reliable protein architectures (marked 'c' Figure 2) as binary characters. In both cases we were careful to include only characters that were strongly suggested to be monophyletic by the phylogenetic analysis, allowing for some secondary loss of domain architectures within established kinesin families. To further ameliorate patterns of secondary loss we coded the presence and absence of kinesin across the 8 higher taxonomic units (marked on Figures 1 and 2) to produce a matrix of 8 'taxa' and 39 characters. We used a Dollo parsimony analysis method [27] implemented through Phylip 3.68 [28] to assess the ancestral repertoire implied by several alternative eukaryotic topologies, the best scoring Dollo parsimony tree topology (see Figure 3). To further investigate these alternative topologies we used a second coding of the data; in this case we used only the kinesin subfamilies in Additional file 2 (or kinesin families where no subfamilies had been identified), producing a matrix of 8 taxa and 51 characters. Kinesin family member that did not fall into any of the subfamilies were coded as uncertainty in any absences for the other subfamilies.

## Abbreviations

aLRT: approximate likelihood ratio test; HMM: hidden Markov model; IFT: intraflagellar transport; LCEA: last common eukaryotic ancestor; MAR: minimal ancestral repertoire; MCMCMC: metropolis-coupled Markov chain Monte Carlo.

## Authors' contributions

BW and TAR conceived of the study and designed and performed the experiments. BW carried out the phylogenetic analysis of motor domains. TAR carried out the architecture analysis and Dollo analysis. All authors reviewed and interpreted the data. The manuscript was written by BW and TAR. All authors read and approved the final manuscript.

## Supplementary Material

Additional file 1 **HMM-based identification of putative kinesin proteins**. Click here for file

Additional file 2 **Comprehensive phylogenetic analysis of the kinesin protein superfamily.** Bayesian phylogeny of 1263 kinesins from 45 diverse eukaryotes. Click here for file

Additional file 3 All 1624 putative kinesin sequences recovered with results of Pfam and CDD analysis and paralogue family/subfamily classification. Click here for file

Additional file 4 Diversity of kinesin protein domain architectures. Click here for file

Additional file 5 Identification of stable kinesin domain architectural characters for ancestral kinesin repertoire analysis. Click here for file

Additional file 6 Tests for homology of additional protein domains between distantly related kinesins with similar protein domain architectures. Click here for file

Additional file 7 List of sources and versions of predicted protein datasets from 45 eukaryotes used in this work. Click here for file

Additional file 8 Fasta file containing full alignment of kinesin motor domains from 1458 sequences passing the inclusion threshold. Click here for file

Additional file 9 Fasta file containing trimmed, reduced-redundancy alignment of 1263 sequences used for phylogenetic inference. Click here for file

Additional file 10 Comparison of performance of aLRT results and Bayesian posterior probabilities. Click here for file

Additional file 11 Distribution of kinesin motor domain 'quality' as a function of kinesin family membership. Click here for file
