# Supplementary material for: Patterns of kinesin evolution reveal a complex ancestral eukaryote with a multifunctional cytoskeleton
Source: BMC Evol Biol. 2010 Apr 27;10:110. doi: 10.1186/1471-2148-10-110 (PMC2867816; doi:10.1186/1471-2148-10-110)

Additional data file 1 - HMM-based identification of putative kinesin proteins.

The ‘quality’ of the kinesin motor domains in the predicted proteomes of 45 diverse eukaryotes as assessed by match to Pfam motor domain profile PF00225 (see Materials and methods). Matches with an e-value <100 are shown. Dashed lines indicate the thresholds for inclusion in architecture predictions (Pfam gathering threshold; score=-135) and inclusion in phylogenetic tree inference (score=100).

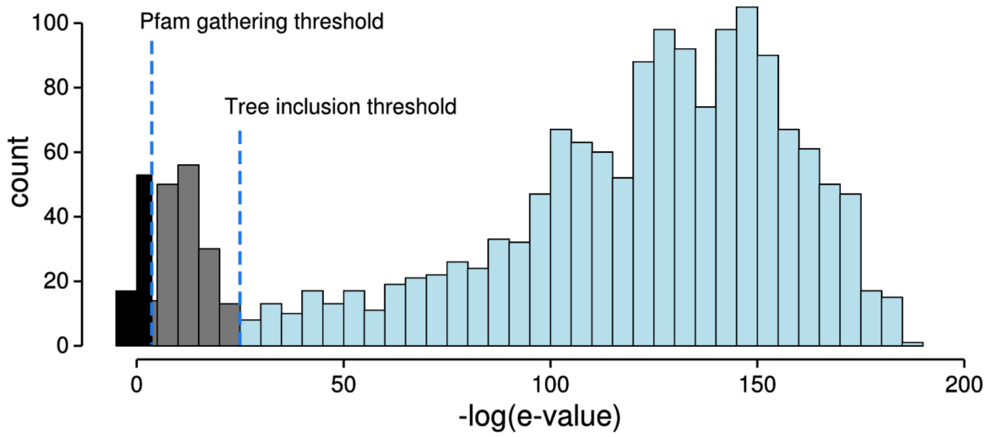

Supplement: Additional file 1 — HMM-based identification of putative kinesin proteins. [file 1471-2148-10-110-S1.PDF]
