# Supplementary material for: Patterns of kinesin evolution reveal a complex ancestral eukaryote with a multifunctional cytoskeleton
Source: BMC Evol Biol. 2010 Apr 27;10:110. doi: 10.1186/1471-2148-10-110 (PMC2867816; doi:10.1186/1471-2148-10-110)
Supplement: Additional file 2 — Comprehensive phylogenetic analysis of the kinesin protein superfamily. Bayesian phylogeny of 1263 kinesins from 45 diverse eukaryotes. [file 1471-2148-10-110-S2.PDF]

Additional data file 2 - Comprehensive phylogenetic analysis of the kinesin protein superfamily. Bayesian phylogeny of 1263 kinesins from 45 diverse eukaryotes.

Majority-rule consensus tree inferred from 8 runs of 12,000,000 generations (see Materials and Methods). Support for the inferred topology from both non-parametric Shimodaira-Hasegawa-like (SH) and parametric χ^2-based (χ^2) approximate-Likelihood Ratio Test is given next to each node (SH/χ^2). Values >0.95 are emboldened. The tree is arbitrarily rooted on an ungrouped kinesin. All identified protein domain architectures are illustrated (not to scale) next to their relevant branch in the phylogeny. Protein sequence names that have been altered from their genome ID to a common name are highlighted (magenta).

Prefixes: Apime: *Apis mellifera*; Arath: *Arabidopsis thaliana*; Auran: *Aureococcus anophagefferens*; Batde: *Batrachochytrium dendrobatidis*; Caeel: *Caenorhabdatis elegans*; Capsp: *Capitella* sp.; Chlre: *Chlamydomonas reinhardtii*; Cioin: *Ciona intestinalis*; Crypa: *Cryptosporidium parvum*; Cyame: *Cyanidioschyzon merolae*; Danre: *Danio rerio*; Dicdi: *Dictyostelium discoideum*; Drome: *Drosophila melanogaster*; Enccu: *Encephalitozoon cuniculi*; Enthi: *Entamoeba histolytica*; Galga: *Gallus gallus*; Giala: *Giardia lamblia*; Homsa: *Homo sapiens*; Leima: *Leishmania major*; Lotgi: *Lottia gigantea*; Monbr: *Monosiga brevicollis*; Naegr: *Naegleria gruberi*; Nemve: *Nematostella vectensis*; Neucr: *Neurospora crassa*; Orysa: *Oryza sativa*; Ostta: *Ostreococcus tauri*; Parte: *Paramecium tetraurelia*; Phatr: *Phaeodactylum tricornutum*; Phypa: *Physcomitrella patens*; Physo: *Phytophthora sojae*; Plafa: *Plasmodium falciparum*; Poptr: *Populus trichocarpa*; Rhior: *Rhizopus oryzae*; Sacce: *Saccharomyces cerevisiae*; Schpo: *Schizosaccharomyces pombe*; Strpu: *Strongylocentrotus purpuratus*; Takru: *Takifugu rubripes*; Tetth: *Tetrahymena thermophila*; Thaps: *Thalassiosira pseudonana*; Thean: *Theileria annulata*; Toxgo: *Toxoplasma gondii*; Triva: *Trichomonas vaginalis*; Triad: *Trichoplax adhaerens*; Trybr: *Trypanosoma brucei*; Ustma: *Ustilago maydis*.

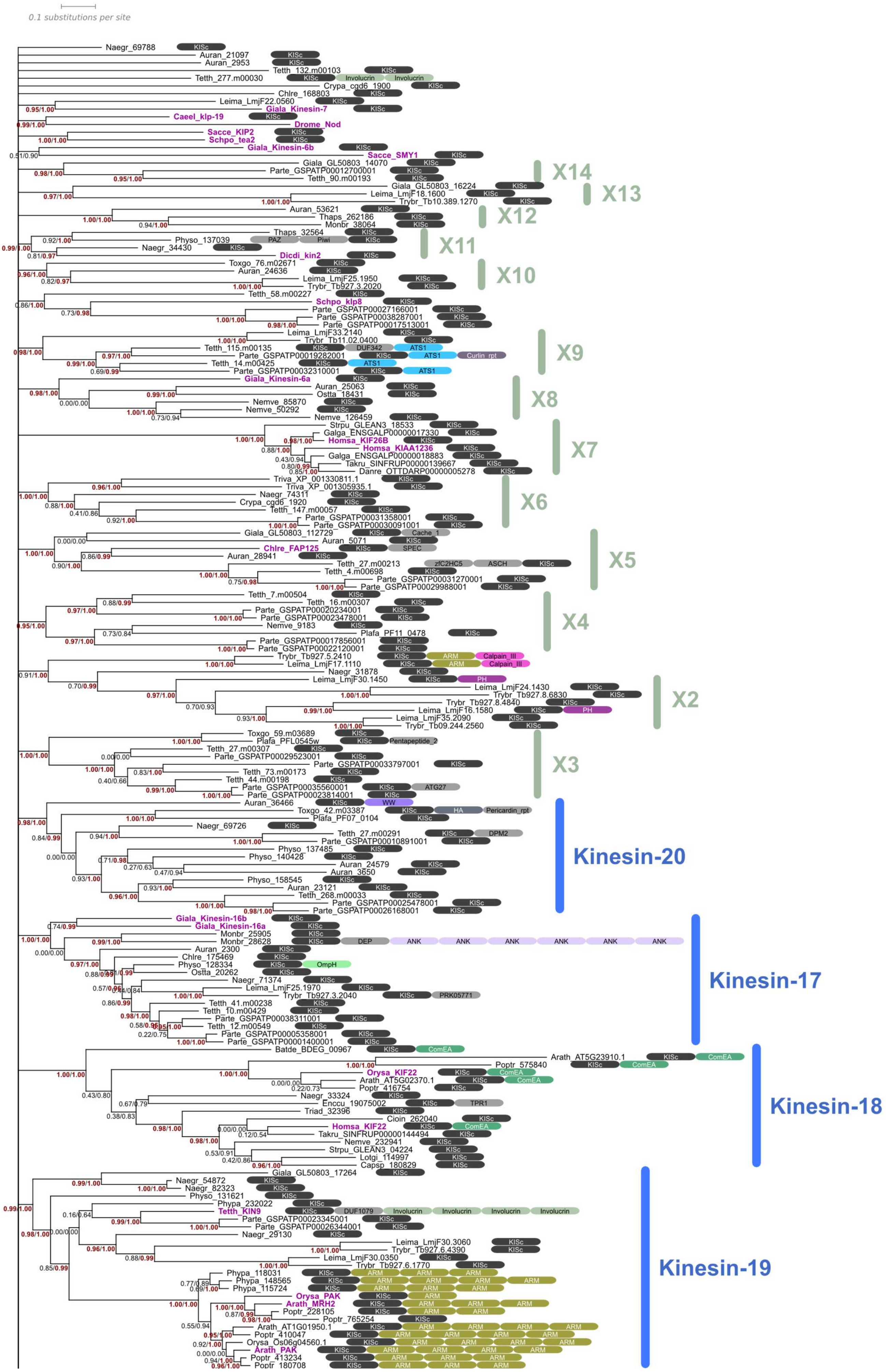

Figure S2: part 1 of 8

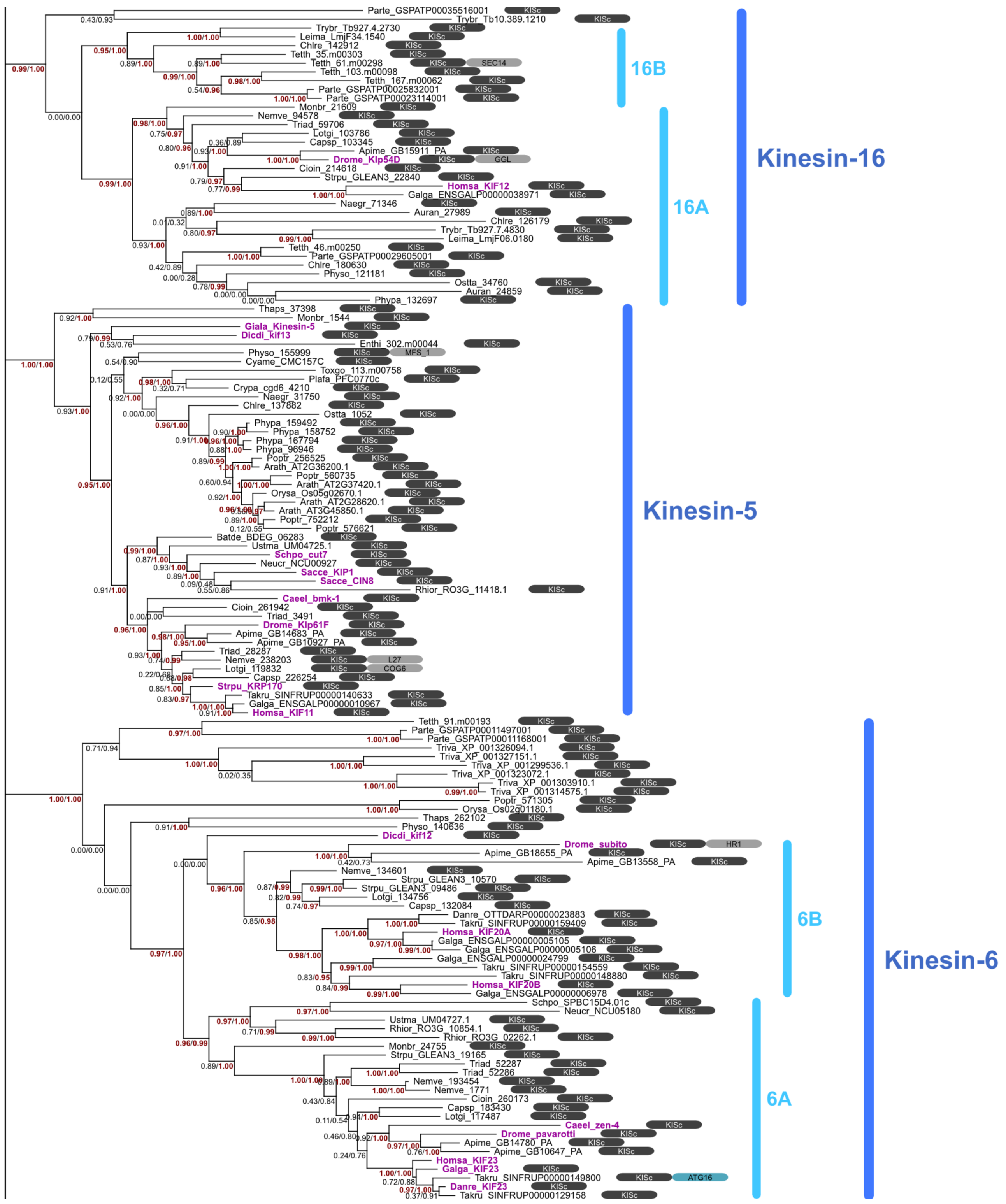

Figure S2: part 2 of 8

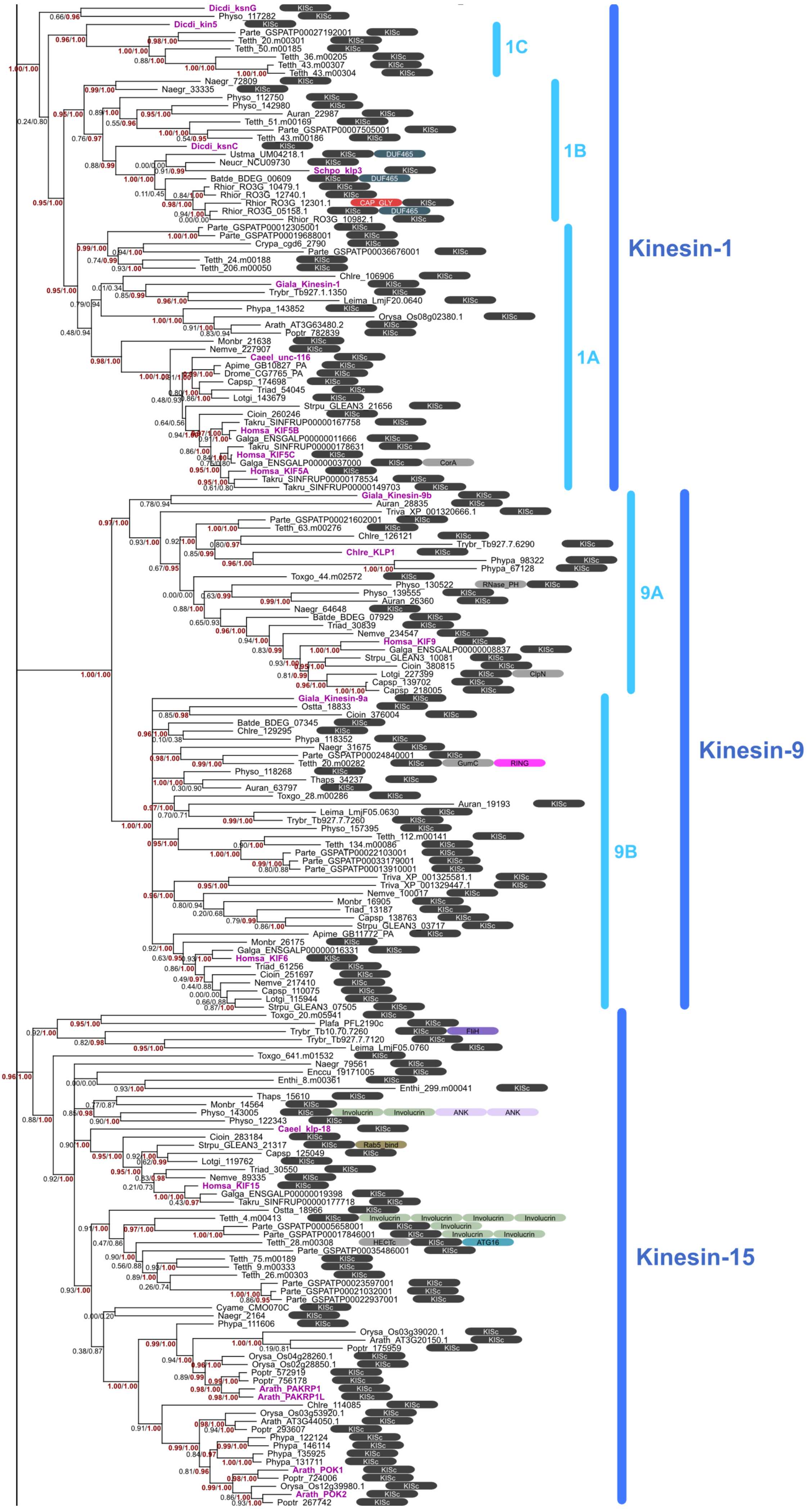

Figure S2: part 3 of 8

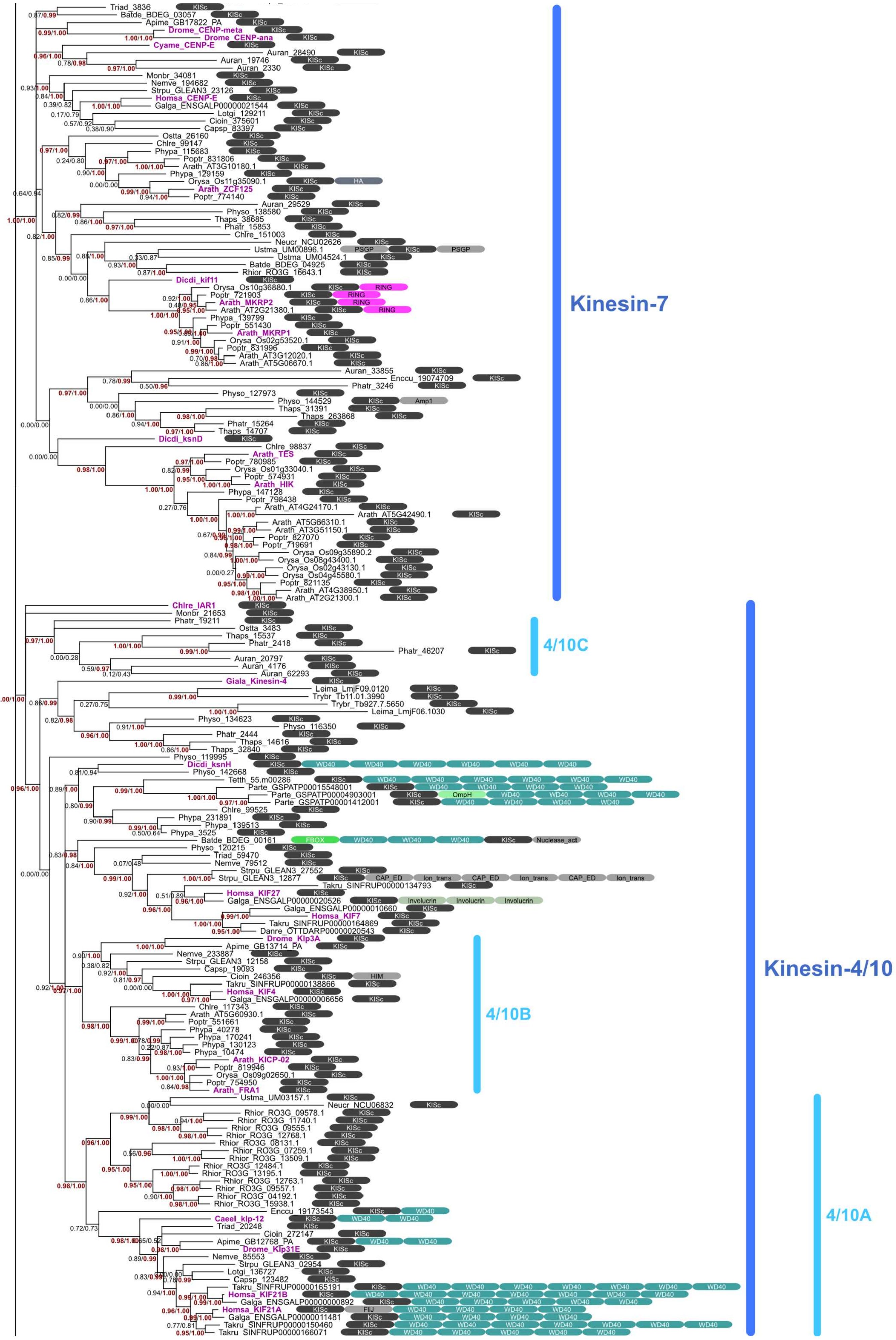

Figure S2: part 4 of 8

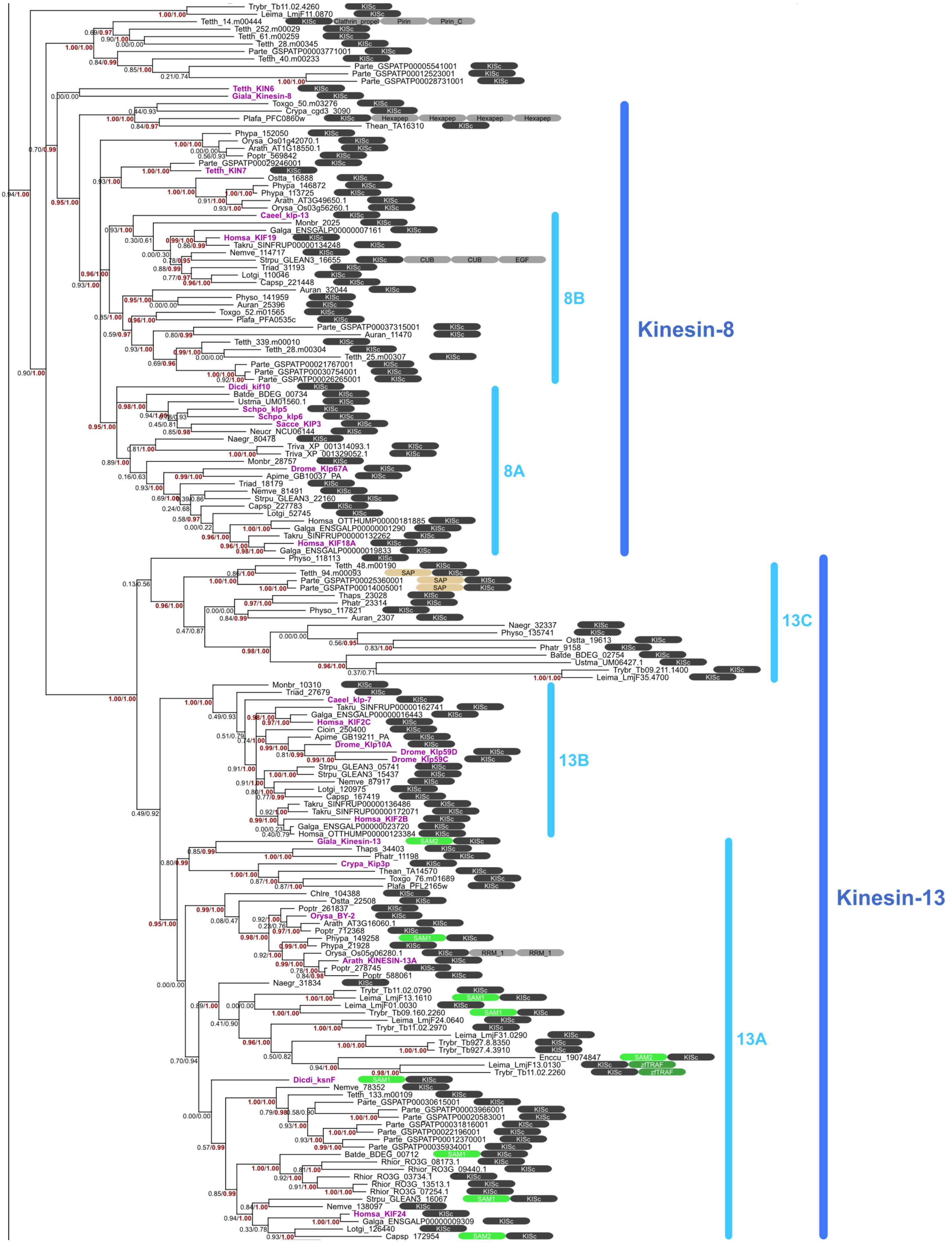

Figure S2: part 5 of 8

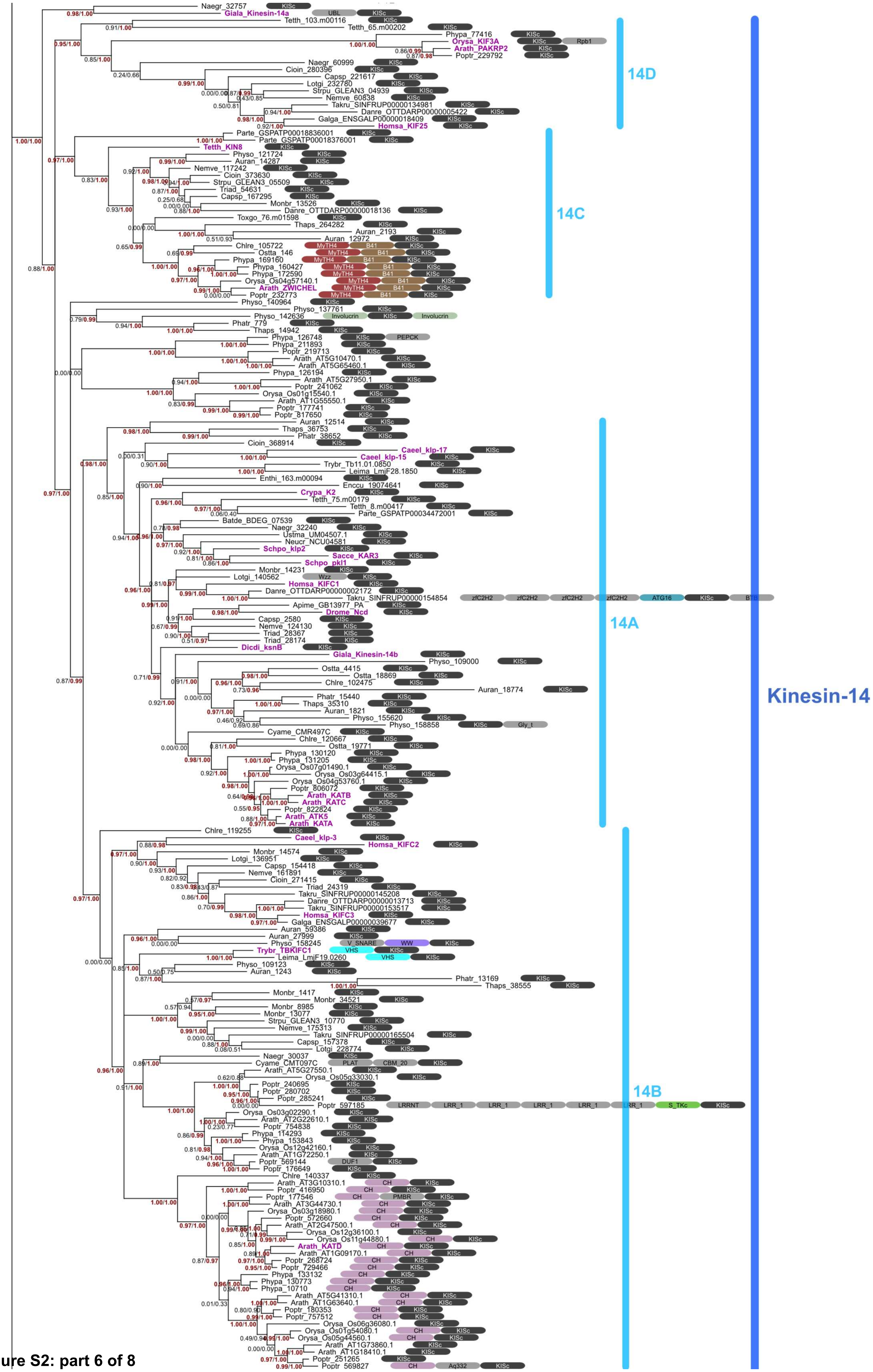

Figure S2: part 6 of 8

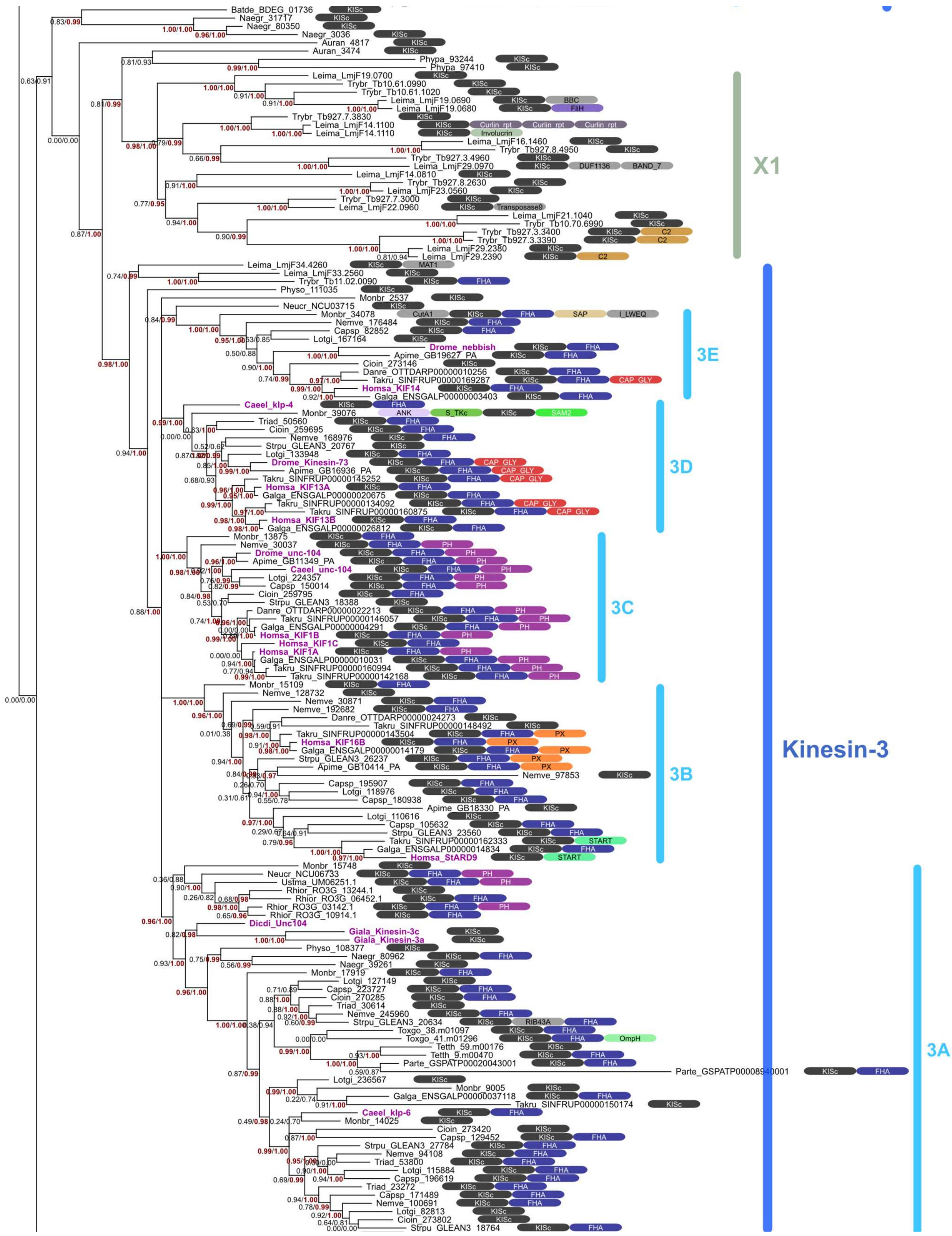

Figure S2: part 7 of 8

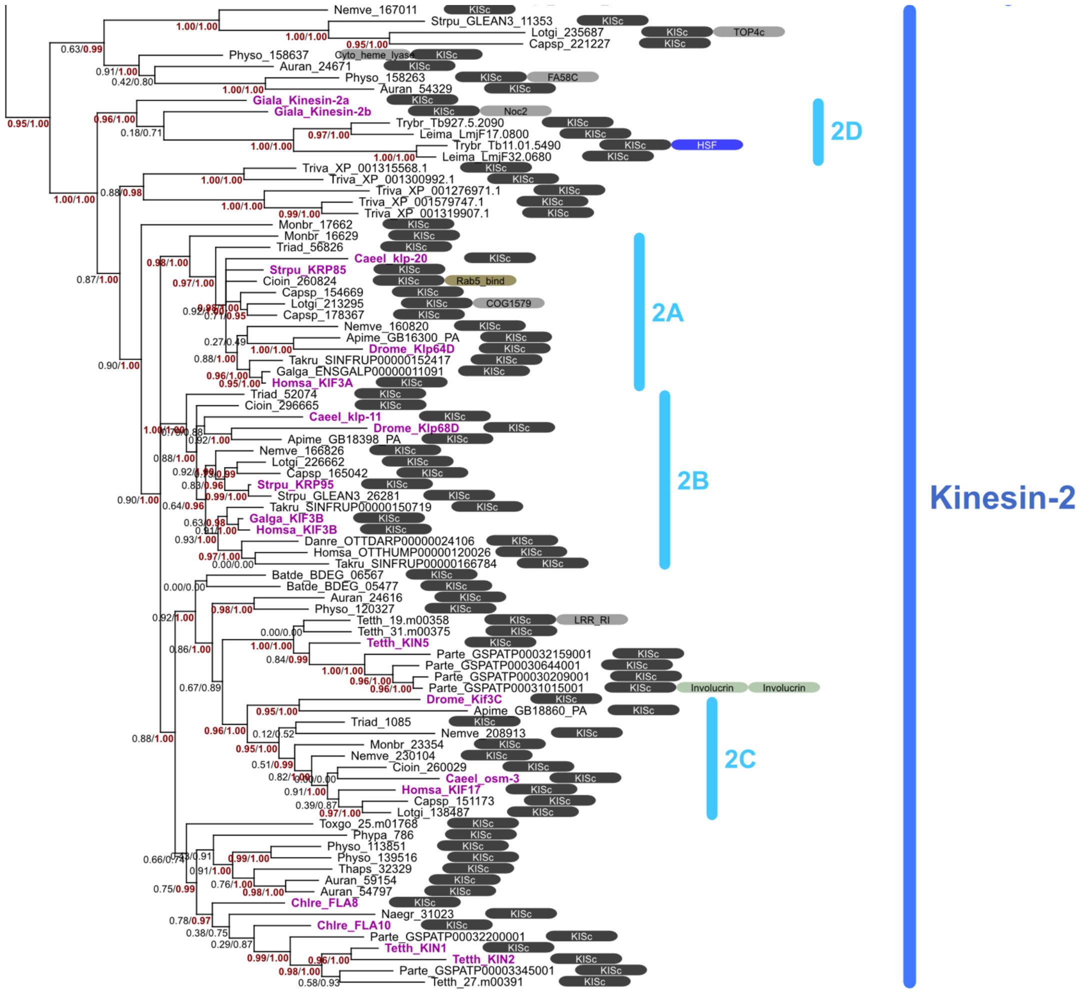

Figure S2: part 8 of 8
