# Supplementary material for: Patterns of kinesin evolution reveal a complex ancestral eukaryote with a multifunctional cytoskeleton
Source: BMC Evol Biol. 2010 Apr 27;10:110. doi: 10.1186/1471-2148-10-110 (PMC2867816; doi:10.1186/1471-2148-10-110)
Supplement: Additional file 4 — Diversity of kinesin protein domain architectures. [file 1471-2148-10-110-S4.PDF]

Additional data file 4 - Diversity of kinesin protein domain architectures.

Pfam and CDD searches were used to identify putative gene architectures for all 1624 putative kinesin proteins and mapped the relative taxon distribution. The domain architectures are noted adjacent to the last column using short domain names. The species are labelled across the top and are grouped into higher taxonomic units. The taxon distribution of architecture characters used for the parsimony tree analyses of the terminal branches of the eukaryotes are marked c (character) adjacent to the first column. While and characters excluded because they were not homologous are marked d(ex) (discounted and excluded) and taxon distributions that were altered with one or more taxa discounted because the architectural character is not homologous are marked d/c (discounted – amended and then counted as a character).

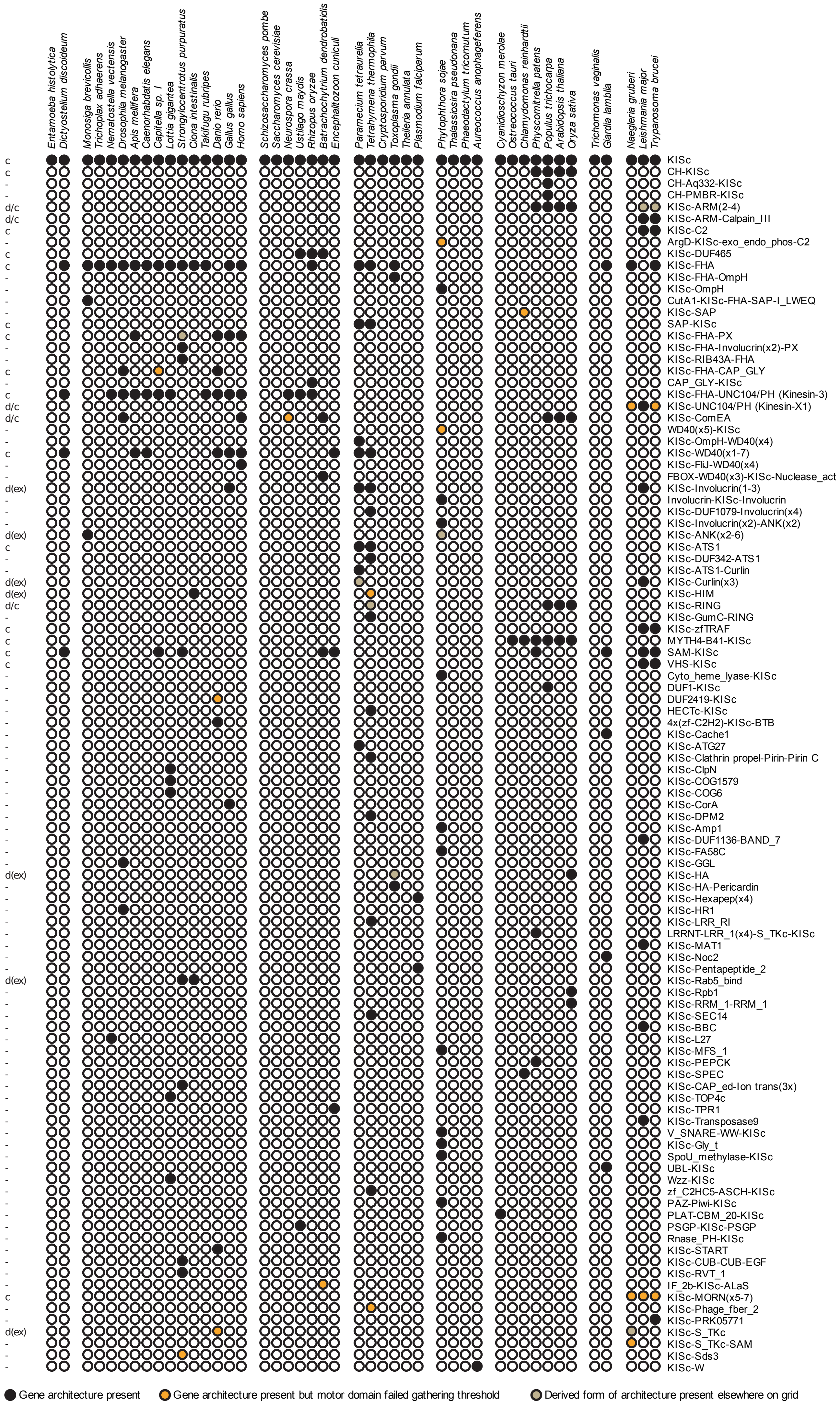
Entamoeba histolytica
Dictyostelium discoideum
Monosiga brevicollis
Trichoplax adhaerens
Nematostella vectensis
Drosophila melanogaster
Apis mellifera
Caenorhabditis elegans
Capitella sp. I
Lottia gigantea
Strongylocentrotus purpuratus
Ciona intestinalis
Takifugu rubripes
Danio rerio
Gallus gallus
Homo sapiens
Schizosaccharomyces pombe
Saccharomyces cerevisiae
Neurospora crassa
Ustilago maydis
Rhizopus oryzae
Batrachochytrium dendrobatidis
Encephalitozoon cuniculi
Paramecium tetraurelia
Tetrahymena thermophila
Cryptosporidium parvum
Toxoplasma gondii
Theileria annulata
Plasmodium falciparum
Phytophthora sojae
Thalassiosira pseudonana
Phaeodactylum tricornutum
Aureococcus anophageferens
Cyanidioschyzon merolae
Ostreococcus tauri
Chlamydomonas reinhardtii
Physcomitrella patens
Populus trichocarpa
Arabidopsis thaliana
Oryza sativa
Trichomonas vaginalis
Giardia lamblia
Naegleria gruberi
Leishmania major
Trypanosoma brucei
c KISc
c CH-KISc
- CH-Aq332-KISc
- CH-PMBR-KISc
d/c KISc-ARM(2-4)
d/c KISc-ARM-Calpain_III
c KISc-C2
- ArgD-KISc-exo_endo_phos-C2
c KISc-DUF465
c KISc-FHA
- KISc-FHA-OmpH
- KISc-OmpH
- CutA1-KISc-FHA-SAP-I_LWEQ
- KISc-SAP
c SAP-KISc
c KISc-FHA-PX
- KISc-FHA-Involucrin(x2)-PX
- KISc-RIB43A-FHA
c KISc-FHA-CAP_GLY
- CAP_GLY-KISc
c KISc-FHA-UNC104/PH (Kinesin-3)
d/c KISc-UNC104/PH (Kinesin-X1)
d/c KISc-ComEA
- WD40(x5)-KISc
- KISc-OmpH-WD40(x4)
c KISc-WD40(x1-7)
- KISc-FliJ-WD40(x4)
- FBOX-WD40(x3)-KISc-Nuclease_act
d(ex) KISc-Involucrin(1-3)
- Involucrin-KISc-Involucrin
- KISc-DUF1079-Involucrin(x4)
- KISc-Involucrin(x2)-ANK(x2)
d(ex) KISc-ANK(x2-6)
c KISc-ATS1
- KISc-DUF342-ATS1
- KISc-ATS1-Curlin
d(ex) KISc-Curlin(x3)
d(ex) KISc-HIM
d/c KISc-RING
- KISc-GumC-RING
c KISc-zfTRAF
c MYTH4-B41-KISc
c SAM-KISc
c VHS-KISc
- Cyto_heme_lyase-KISc
- DUF1-KISc
- DUF2419-KISc
- HECTc-KISc
- 4x(zf-C2H2)-KISc-BTB
- KISc-Cache1
- KISc-ATG27
- KISc-Clathrin propel-Pirin-Pirin C
- KISc-ClpN
- KISc-COG1579
- KISc-COG6
- KISc-CorA
- KISc-DPM2
- KISc-Amp1
- KISc-DUF1136-BAND_7
- KISc-FA58C
- KISc-GGL
d(ex) KISc-HA
- KISc-HA-Pericardin
- KISc-Hexapep(x4)
- KISc-HR1
- KISc-LRR_RI
- LRRNT-LRR_1(x4)-S_TKc-KISc
- KISc-MAT1
- KISc-Noc2
- KISc-Pentapeptide_2
d(ex) KISc-Rab5_bind
- KISc-Rpb1
- KISc-RRM_1-RRM_1
- KISc-SEC14
- KISc-BBC
- KISc-L27
- KISc-MFS_1
- KISc-PEPCK
- KISc-SPEC
- KISc-CAP_ed-Ion trans(3x)
- KISc-TOP4c
- KISc-TPR1
- KISc-Transposase9
- V_SNARE-WW-KISc
- KISc-Gly_t
- SpoU_methylase-KISc
- UBL-KISc
- Wzz-KISc
- zf_C2HC5-ASCH-KISc
- PAZ-Piwi-KISc
- PLAT-CBM_20-KISc
- PSGP-KISc-PSGP
- Rnase_PH-KISc
- KISc-START
- KISc-CUB-CUB-EGF
- KISc-RVT_1
- IF_2b-KISc-ALaS
c KISc-MORN(x5-7)
- KISc-Phage_fber_2
- KISc-PRK05771
d(ex) KISc-S_TKc
- KISc-S_TKc-SAM
- KISc-Sds3
- KISc-W
Gene architecture present
Gene architecture present but motor domain failed gathering threshold
Derived form of architecture present elsewhere on grid
