# Supplementary material for: Patterns of kinesin evolution reveal a complex ancestral eukaryote with a multifunctional cytoskeleton
Source: BMC Evol Biol. 2010 Apr 27;10:110. doi: 10.1186/1471-2148-10-110 (PMC2867816; doi:10.1186/1471-2148-10-110)
Supplement: Additional file 5 — Identification of stable kinesin domain architectural characters for ancestral kinesin repertoire analysis. [file 1471-2148-10-110-S5.PDF]

Additional data file 5 - Identification of stable kinesin domain architectural characters for ancestral kinesin repertoire analysis.

Domain Combination	Phylogenetic Position
CH-KISc	Monophyletic cluster with some evidence of secondary loss within 14B
KISc-ARM(2-4)	Monophyletic cluster with some evidence of secondary loss within K19
KISc-ARM-Calpain_III	Monophyletic – branches separately from kinesin families
KISc-C2	Monophyletic cluster within X1
KISc-DUF465	Monophyletic cluster with some evidence of secondary loss within K1B
KISc-FHA	Monophyletic cluster with some evidence of secondary loss within K3
SAP-KISc	Monophyletic cluster with some evidence of secondary loss within K13C
KISc-FHA-PX	Monophyletic cluster with some evidence of secondary loss within K3B
KISc-FHA-CAP_GLY	Monophyletic cluster with some evidence of secondary loss within K3 (D/E)
KISc-FHA-UNC104/PH	Monophyletic cluster with some evidence of secondary loss within K3
KISc-UNC104/PH	Monophyletic cluster with some evidence of secondary loss within X2
KISc-ComEA	Monophyletic cluster with some evidence of secondary loss within K18 with one additional case in *Drosophila* currently not assigned to a family
KISc-WD40(x1-7)	Monophyletic cluster with evidence of extensive secondary loss within K4/10
KISc-ATS1	Monophyletic cluster within X9
KISc-RING	The plant KISc-RING protein architecture appears to be a monophyletic cluster within K7, the *Tetrahymena* KISc-GUMC-RING protein is excluded as a separate character see Table S2
KISc-zfTRAF	Monophyletic cluster within K13A
MYTH4-B41-KISc	Monophyletic cluster within K14C
SAM-KISc	Monophyletic cluster with evidence of extensive secondary loss within K13A
VHS-KISc	Monophyletic cluster within K14B
KISc-MORN(x5-7)	Not included in phylogeny divergent kinesin motor domain but this architecture is only found in closely related kinetoplastid taxa.
