# Supplementary material for: Patterns of kinesin evolution reveal a complex ancestral eukaryote with a multifunctional cytoskeleton
Source: BMC Evol Biol. 2010 Apr 27;10:110. doi: 10.1186/1471-2148-10-110 (PMC2867816; doi:10.1186/1471-2148-10-110)
Supplement: Additional file 6 — Tests for homology of additional protein domains between distantly related kinesins with similar protein domain architectures. [file 1471-2148-10-110-S6.PDF]

**Additional data file 6 - Tests for homology of additional protein domains between distantly related kinesins with similar protein domain architectures.**

We identified several kinesin domain architectures, which include domains present in a low number of distantly related genomes or for which the kinesin motor domains belong to distantly related paralogue families. In these cases we conducted further analysis to investigate whether these sequences were composed of domains related by either convergence or vertical inheritance, or if the domain classification was artefactual. For each candidate domain architecture, marked ‘d’ on Figure 2, functional and annotation data was accessed and domain alignments were made. We identified 10 cases of domain classification with the same domain name, but apparently non-homologous sequences that are therefore likely to be artefact and either excluded or adjusted the taxon distribution of this character. Additionally, we found that the SAM1 and SAM2 domains are homologous and are therefore both classified as SAM for the purposes of this study.

| Domain | Species | Exclusions of architectures (d/ex) / corrections of architecture distribution (d/c) / corrections in annotations | Results of PFAM, CDD and alignment analysis |
|---|---|---|---|
| KISc-ARM<br>KISc-ARM-Calpain_III | *Physcomitrella patens*<br>*Populus trichocarpa*<br>*Arabidopsis thaliana*<br>*Oryza sativa*<br>*Leishmania major*<br>*Trypanosoma brucei* | Corrections of architecture distribution (plant and kinetoplastid groups treated separately) | Armadillo/beta-catenin-like repeat of 40 amino acids. Its composed of tandem repeats that form a super-helix of helices that is proposed to mediate interaction of beta catenin and is involved in signal transduction, cytoskeletal regulation, and other intracellular signalling in mammals [1,2]. Alignment of the kinetoplastid and plant kinesin ARM domains revealed a very low level of sequence homology between the two divergent taxonomic groups. Furthermore, this putative kinesin sequence character was found to group into two divergent Kinesin paralogue groups and into two different protein architectural forms. We therefore find this domain is unreliable as a monophyletic derived kinesin character and therefore have counted the two architectures separately the plant KISc-ARM and the kinetoplastid KISc-ARM-Calpain_III (marked d/c on Figure 2 and Figure S3). |

| | | | |
|---|---|---|---|
| KISc-PH/UNC104<br>KISc-FHA-PH/UNC104 | *Naegleria gruberi*<br>*Leishmania major*<br>*Trypanosoma brucei*<br>*Dictyostelium discoideum*<br>*Nematostella vectensis*<br>*Drosophila melanogaster*<br>*Apis mellifera*<br>*Caenorhabditis elegans*<br>*Capitella sp.*<br>*Lottia gigantea*<br>*Danio rerio*<br>*Takifugu rubripes*<br>*Gallus gallus*<br>*Homo sapiens*<br>*Neurospora crassa*<br>*Ustilago maydis*<br>*Rhizopus oryzae* | Corrections of architecture distribution based on membership to kinesin-3 and kinesin-X1 (see Figure 2) | The KISc-PH/UNC104 protein domain (~100 amino acids) architectures were present in two separate and unrelated kinesin paralogue families and with two distinct protein domain architectures (KISc-PH/UNC104 and KISc-FHA-PH/UNC104) suggesting the fusion of the PH/UNC104 domain to a kinesin motor domain occurred separately in two distantly related kinesin paralogues. Alignment of the ~100 amino acid kinesin-3 and kinesin-X1 PH/UNC104 domain region showed very low identifiable alignment similarity even when we included additional PH/UNC104 domain containing protein sequences from GenBank. This prevent us from using phylogenies to test the ancestry of the kinesin-3 and kinesin-X1 PH/UNC104 domains and suggesting that one of these domains may be a false positive CDD/PFAM domain identification or that they are very divergent representatives of this protein domain family and that this domain architecture evolved by convergence. These similar but unrelated PH/UNC104 architectural forms were both present in multiple species and were therefore counted separately in our model of ancient kinesin protein evolution (marked d/c on Figure 2 and Figure S3). |
| KISc-Involucrin_rpt | *Paramecium tetraurelia*<br>*Leishmania major*<br>*Phytophthora sojae*<br>*Strongylocentrotus purpuratus*<br>*Tetrahymena thermophila*<br>*Gallus gallus* | Exclusions of architectures | Involucrin repeats are short protein motif which PFAM HMM identifies as a ~10 amino acid characters. The domain has a core conserved QxxQ amino acid motif [1,2]. Based on the small size of this protein domain and the low level of conserved sequence characteristics across the different kinesin Involucrin repeat domains it is difficult to demonstrate that they are homologues and have not evolved convergently. Furthermore, this putative kinesin sequence character was consistently found scattered across different kinesin paralogue families and distantly related taxa. We therefore find this domain unreliable as a monophyletic derived kinesin character. |
| KISc-ANK | *Monosiga brevicollis*<br>*Phytophthora sojae* | Exclusions of architectures | Ankyrin repeats occur in a large number of functionally diverse proteins mainly from eukaryotes. The repeat has been found in proteins of diverse function such as transcriptional initiators, cell-cycle regulators, cytoskeletal, ion transporters and signal transducers. Ankyrin repeats are tandemly repeated modules of about 33 amino acids. The repeats associate to form a higher order structure and there is generally no clear separation between noise and signal on the HMM search [1,2]. This combined with their short sequence length and that the two KISc-ANK domain arrangements detected were found in unrelated phylogenetic groups suggests that this domain character is unreliable as a monophyletic derived kinesin character. |

| | | | |
|---|---|---|---|
| KISc-Curlin_rpt | *Leishmania major*<br>*Paramecium tetraurelia* | Exclusions of architectures | Repeat motif of about 30 residues often associated with curli fibres which are thin aggregative fibres involved in adhesion; binding lamin, fibronectin, plasminogen, human contact phase proteins, and MHC class I molecules [1,2]. The *Leishmania major* kinesin had three partial curlin repeat domains, all three having three identical 8 amino acid motif similar to the c-termini of the curlin repeat domain HMM. The *Paramecium* kinesin partial curlin repeat domain has 11 amino acid motif similar to the c-termini of the curlin repeat domain HMM [1,2]. Based on the small size of this protein domain and the low level of conserved sequence characteristics it is difficult to demonstrate that the *Paramecium* and *Leishmania* curlin repeat domain are homologues. Furthermore, as these two kinesins group in separate kinesin paralogue families it is unlikely that they are a monophyletic derived kinesin character. |
| KISc-HIM | *Ciona intestinalis*<br>*Tetrahymena thermophila* | Exclusions of architectures | This short motif (~25 amino acid residues) is often found in invasins and haemagglutinins proteins [1,2]. Alignment of the *Ciona* and *Tetrahymena* kinesin HIM domains revealed a very low level of sequence homology between the two divergent taxonomic groups. Furthermore, this putative kinesin sequence character was found in separate parts of the kinesin phylogeny and in two different protein architectural forms. We therefore find this domain unreliable as a monophyletic derived kinesin character. |
| KISc-RING | *Populus trichocarpa*<br>*Arabidopsis thaliana*<br>*Oryza sativa*<br>*Tetrahymena thermophila* | Corrections of architecture distribution (*Tetrahymena* excluded) | Ring domains are a specialized type of Zn-finger domain and are composed of a variable region of 40-60 amino acids with cysteine/histidine pattern forming a 'cross brace' [1,2]. Sequence similarity between plant and ciliate kinesin Ring domains are very low and the kinesin motor domain branches within different distantly related paralogues and with different protein architectures suggesting that the two architectural forms are unrelated. However, the plant KISc-RING domain architecture appears to be a monophyletic anciently derived character and is therefore included in our ancestral model analysis (marked d/c on Figure 2 and Figure S3). |
| SAM (1/2)-KISc | *Physcomitrella patens*<br>*Strongylocentrotus purpuratus*<br>*Trypanosoma brucei*<br>*Batrachochytrium dendrobatidis*<br>*Capitella sp.*<br>*Dictyostelium discoideum*<br>*Encephalitozoon cuniculi*<br>*Giardia lamblia*<br>*Leishmania major* | Correction of annotation name | SAM domains (Sterile alpha motifs) are ~60 amino acid protein modules involved in a range of diverse protein-protein interactions [1,2]. Alignment of the Kinesin SAM1 and SAM2 domains suggests that both sub-classes include divergent amino acid sequences and demonstrate no distinguishable amino acid alignment motifs, suggesting the two classes are related. Both SAM1-KISc and SAM2-KISc fall into the same Kinesin paralogue group (Kinesin 13). As such we have adopted the CDD classification system that does not separate the SAM domain into SAM1 or SAM2 subclasses because it is unlikely that these two domains represent distinct kinesin characters. |

| | | | |
|---|---|---|---|
| KISc-HA | *Oryza sativa*<br>*Toxoplasma gondii* | Exclusions of architectures | This short domain is found in multiple copies in bacterial helicase proteins. The domain of about 75 amino acids is predicted to contain 3 alpha helices. The function of this domain may be to bind nucleic acid [1,2]. The two putative HA domains found in combination with the KISc domains were partial [1,2]. The *Oryza* domain has weak similarity to the first 21 amino acids of the HA domain HMM while the *Toxoplasma* domain has similarity to the last 26 amino acids of the HA domain HMM. Furthermore, this putative kinesin sequence character was found in separate parts of the kinesin phylogeny and in two different protein architectural forms. We therefore find this domain unreliable as a monophyletic derived kinesin character. |
| KISc-RAB5_Bind | *Strongylocentrotus purpuratus*<br>*Ciona intestinalis* | Exclusions of architectures | This domain architecture appears in the genome of two closely related animals but the kinesin proteins belong to two distantly related paralogues, Kinesin 15 and Kinesin 2, suggesting that this domain architecture has evolved independently as a case of convergent evolution. Alignment of the RAB5_Bind domain amino acid sequences (~100 amino acids) suggests that the two RAB5_Bind domains are highly divergent with very little amino acid alignment similarity. This prevented us from using phylogenetic analysis to investigate the evolutionary ancestry of the kinesin RAB5-bind domains and suggested that one of these domains may be a false positive CDD/PFAM domain identification, or that they are very divergent representatives of the RAB5-bind domain and that this kinesin architecture evolved by convergence. |
| KISc-S_TKc | *Naegleria gruberi*<br>*Takifugu rubripes* | Exclusions of architectures | The S_TKc domain is recognised by PFAM as a kinase domain, which include a highly diverse family of protein domains [1,2]. The *Takifugu* KISc-S_TKc domain architecture contains only a short ~30 amino acid fragment of the kinase HMM model. These kinesin architectures were found in separate parts of the kinesin phylogeny and in two different protein architectural forms. We therefore find this domain unreliable as a monophyletic derived kinesin character. |

**References**


1. Bateman A, Coin L, Durbin R, Finn RD, Hollich V, et al. (2004) The Pfam protein families database. Nucleic Acids Res 32: D138-141.
2. Marchler-Bauer A, Anderson JB, Cherukuri PF, DeWeese-Scott C, Geer LY, et al. (2005) CDD: a Conserved Domain Database for protein classification. Nucleic Acids Res 33: D192-196.
3. Galtier N, Gouy M, Gautier C (1996) SEAVIEW and PHYLO_WIN: two graphic tools for sequence alignment and molecular phylogeny. Comput Appl Biosci 12: 543-548.
4. Edgar RC (2004) MUSCLE: a multiple sequence alignment method with reduced time and space complexity. BMC Bioinformatics 5: 113.
