# Supplementary material for: Patterns of kinesin evolution reveal a complex ancestral eukaryote with a multifunctional cytoskeleton
Source: BMC Evol Biol. 2010 Apr 27;10:110. doi: 10.1186/1471-2148-10-110 (PMC2867816; doi:10.1186/1471-2148-10-110)
Supplement: Additional file 7 — List of sources and versions of predicted protein datasets from 45 eukaryotes used in this work. [file 1471-2148-10-110-S7.PDF]

Additional data file 7 - List of sources and versions of predicted protein datasets from 45 eukaryotes used in this work.

Organism	Source	Version	Web reference	Download filename
Apis mellifera	beebase	v2 (prerelease)	racerx00.tamu.edu	Amel_pre_release2_OGS_pep.fa
Arabidopsis thaliana	TAIR	TAIR7	www.arabidopsis.org/	TAIR7_pep_20070425
Aureococcus anophagefferens	JGI	v1.0	www.jgi.doe.gov/	proteins.Auran1_FilteredModels3.fasta.gz
Batrachochytrium dendrobatidis	Broad Institute	v1.0	www.broad.mit.edu/annotation/fgi/	batrachochytrium_dendrobatidis_1_proteins.fasta.gz
Caenorhabdatis elegans	WormBase	WS170	www.wormbase.org/	wormpep170.tar.gz
Capitella sp. I	JGI	v1.0	www.jgi.doe.gov/	FilteredModelsv1.0.aa.fasta.gz
Chlamydomonas reinhardtii	JGI	v3.1	www.jgi.doe.gov/	Chlre3_1.GeneCatalogProteins.6JUL06.fasta.gz
Ciona intestinalis	JGI	v2.0	www.jgi.doe.gov/	FM1.aa.fasta.gz
Cryptosporidium parvum	CryptoDB	v3.4	www.cryptodb.org/	CparvumAnnotatedProtein.fsa.zip
Cyanidioschyzon merolae	C.merolae genome project	?	merolae.biol.s.u-tokyo.ac.jp/	cds.fasta
Danio rerio	VEGA	May 2007	vega.sanger.ac.uk/	Danio_rerio.VEGA.may.pep.tot.fa.gz
Dictyostelium discoideum	dictyBase	05-20-2007	dictybase.org/	dicty_predictions_protein.gz
Drosophila melanogaster	ENSEMBL	BDGP4.3, 44.43b	www.ebi.ac.uk/ensembl/	Drosophila_melanogaster.BDGP4.3.44.pep.all.fa.gz
Encephalitozoon cuniculi	NCBI	?	www.ncbi.nlm.nih.gov	encephalitozoon cuniculi Field: Organism
Entamoeba histolytica	geneDB	17102005	www.genedb.org/	GeneDB_Ehistolytica_Proteins_17102005
Gallus gallus	ENSEMBL	WASHUC2, 44.2b	www.ebi.ac.uk/ensembl/	Gallus_gallus.WASHUC2.44.pep.all.fa.gz
Giardia lamblia	GiardiaDB	v1.0	www.giardiadb.org/	GlambliaAnnotatedProteins_GiardiaDB-1.0.fasta
Homo sapiens	VEGA+Refseq	'Jun 2007	vega.sanger.ac.uk/, www.ncbi.nlm.nih.gov	Homo_sapiens.VEGA.jun.pep.tot.fa.gz
Leishmania major	geneDB	v5.2	www.genedb.org/	LmjFwholegenome_20060901_V5.2.pep
Lottia gigantea	JGI	v1.0	www.jgi.doe.gov/	Lotgi1_GeneModels_FilteredModels1_aa.fasta.gz
Monosiga brevicollis	JGI	v1.0	www.jgi.doe.gov/	Monbr1_best_proteins.fasta.gz
Naegleria gruberi	JGI	v1.0	www.jgi.doe.gov/	Naegr1_best_proteins.fasta.gz
Nematostella vectensis	JGI	v1.0	www.jgi.doe.gov/	proteins.Nemve1FilteredModels1.fasta.gz
Neurospora crassa	Broad Institute	v7.0	www.broad.mit.edu/annotation/fgi/	neurospora_crassa_7_proteins.fasta.gz

Oryza sativa	TIGR	v5.0	www.tigr.org/tdb/e2k1/osa1/	all.pep
Ostreococcus tauri	JGI	v2.0	www.jgi.doe.gov/	O.tauri.FM.aa.fasta.gz
Paramecium tetraurelia	ParameciumDB	v1.04	paramecium.cgm.cnrs-gif.fr/	Ptetraurelia_peptides_v1.04.fasta
Phaeodactylum tricornutum	JGI	v2.0	www.jgi.doe.gov/	Phatr2_geneModels_FilteredModels2_aa.fasta.gz
Physcomitrella patens	JGI	v1.1	www.jgi.doe.gov/	proteins.Phypa1_1.FilteredModels.fasta.gz
Phytophthora sojae	JGI	v1.1	www.jgi.doe.gov/	proteins.FM_Physo1_1.fasta.gz
Plasmodium falciparum	PlasmoDB	v5.2	www.plasmodb.org/	PfalciparumAnnotatedProteins_plasmoDB-5.2.fasta
Populus trichocarpa	JGI	v1.1	www.jgi.doe.gov/	proteins.Poptr1_1.JamboreeModels.fasta.gz
Rhizopus oryzae	Broad Institute	v3.0	www.broad.mit.edu/annotation/fgi/	rhizopus_oryzae_3_proteins.fasta.gz
Saccharomyces cerevisiae	Saccharomyces genome database	20070713	www.yeastgenome.org/	orf_trans_all.fasta.gz
Schizosaccharomyces pombe	Sanger	v19	www.sanger.ac.uk/Projects/S_pombe	pompep
Strongylocentrotus purpuratus	Baylor College of Medicine	Spur_2.1, v4	www.hgsc.bcm.tmc.edu/	freeze4.tar.gz
Takifugu rubripes	ENSEMBL	Assembly4, 44.4e	www.ebi.ac.uk/ensembl/	Takifugu_rubripes.FUGU4.44.pep.all.fa.gz
Tetrahymena thermophila	TIGR	10/24/2006	www.tigr.org/tdb/e2k1/tta1/	TTA1.pep
Thalassiosira pseudonana	JGI	v3.0	www.jgi.doe.gov/	Thaps3_geneModels_FilteredModels2_aa.fasta.gz
Theileria annulata	GeneDB	v4	www.genedb.org/	TANN.GeneDB.pep
Toxoplasma gondii	ToxoDB	v4.1	www.toxodb.org/	TgondiiAnnotatedProteins_toxoDb-4.1.fasta
Trichomonas vaginalis	TIGR	20050331	www.tigr.org/tdb/e2k1/tvg/	T.vaginalis_20050331.aa.fasta.gz
Trichoplax adhaerens	JGI	v1.0	www.jgi.doe.gov/	Triad1_best_proteins.fasta.gz
Trypanosoma brucei	geneDB	v4	www.genedb.org/	Tb927_Proteins_May06_v4.fas.gz
Ustilago maydis	Broad Institute	v1.0	www.broad.mit.edu/annotation/fgi/	ustilago_maydis_1_proteins.fasta.gz
