# Supplementary material for: Patterns of kinesin evolution reveal a complex ancestral eukaryote with a multifunctional cytoskeleton
Source: BMC Evol Biol. 2010 Apr 27;10:110. doi: 10.1186/1471-2148-10-110 (PMC2867816; doi:10.1186/1471-2148-10-110)

Additional data file 10 - Comparison of performance of aLRT results and Bayesian posterior probabilities.

Comparison shows node support for kinesin phylogeny shown in Additional data file 2 inferred from Bayesian posterior probabilities (PP; 8 runs of 12,000,000 generations) or non-parametric Shimodaira-Hasegawa-like (SH) and parametric χ^2-based (Chi2) approximate Likelihood Ratio Test.

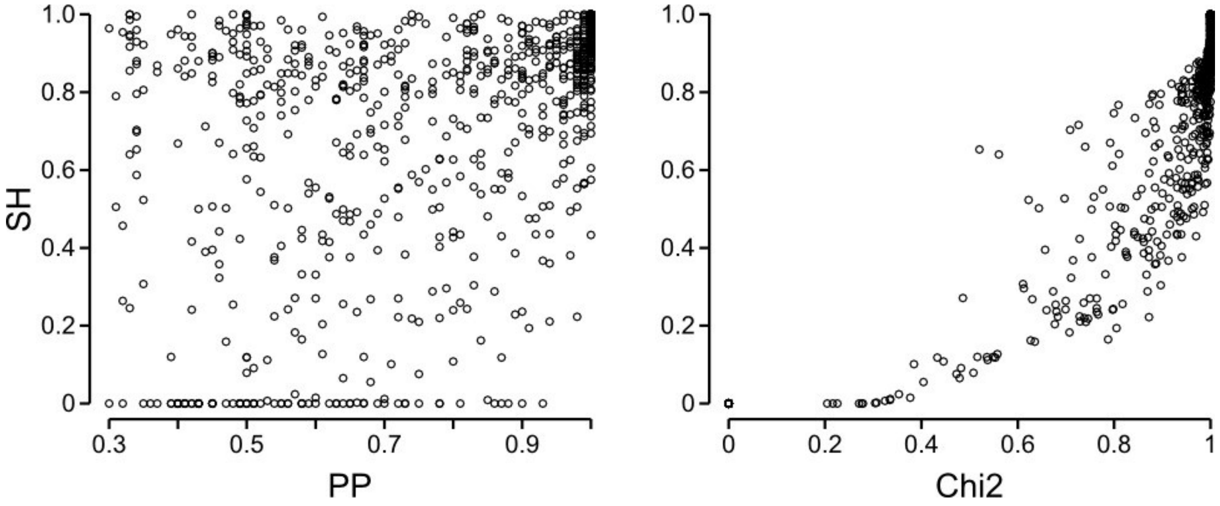

Supplement: Additional file 10 — Comparison of performance of aLRT results and Bayesian posterior probabilities. [file 1471-2148-10-110-S10.PDF]
