# Supplementary material for: Patterns of kinesin evolution reveal a complex ancestral eukaryote with a multifunctional cytoskeleton
Source: BMC Evol Biol. 2010 Apr 27;10:110. doi: 10.1186/1471-2148-10-110 (PMC2867816; doi:10.1186/1471-2148-10-110)

Additional data file 11 - Distribution of kinesin motor domain 'quality' as a function of kinesin family membership.

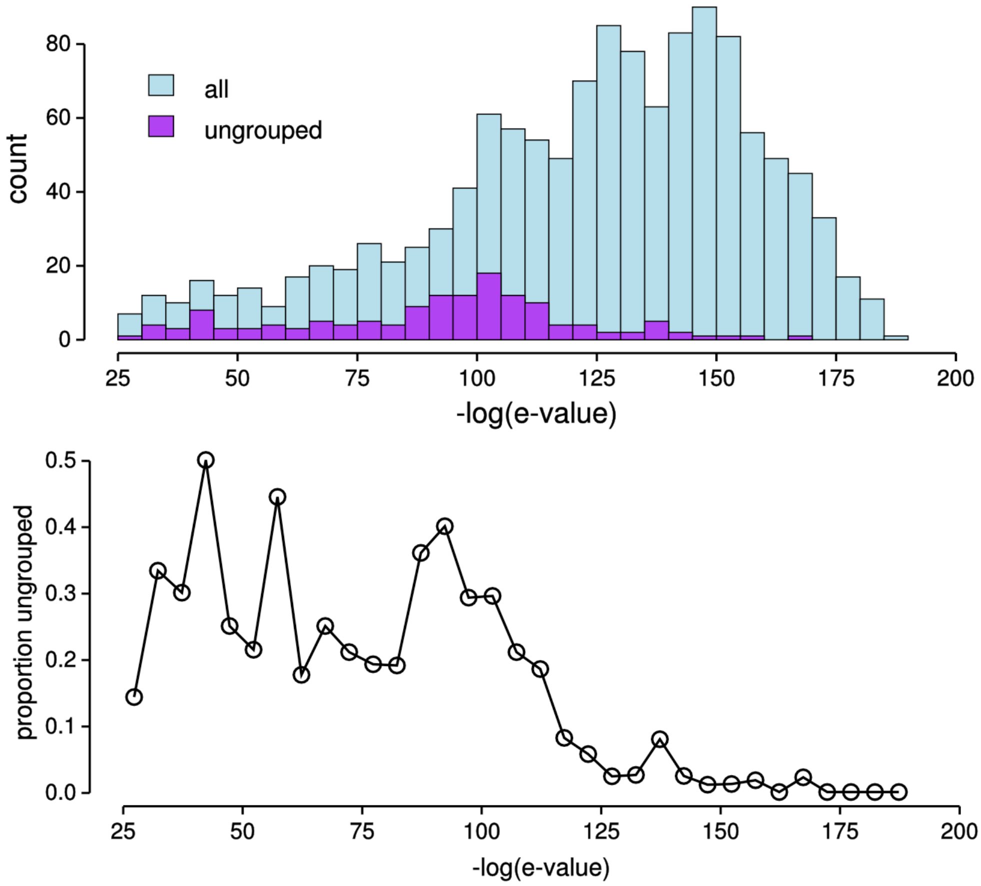

Supplement: Additional file 11 — Distribution of kinesin motor domain 'quality' as a function of kinesin family membership. [file 1471-2148-10-110-S11.PDF]
